# Developing the World‐Wide FINGERS biorepository for dementia risk reduction trials

**DOI:** 10.1002/alz.71949

**Published:** 2026-11-09

**Authors:** Anna Matton, Chinedu Udeh‐Momoh, Makrina Daniilidou, Erika Bereczki, Vilma Alanko, Paola Padilla, Laura D. Baker, Kitti Ban, Miguel G. Borda, Paulo Caramelli, Pablo Martinez‐Lage Alvarez, Marta del Campo, Carlo Ferrarese, Tobias Hartmann, Hamidul Huque, Thomas K. Karikari, Derya Kaya, Rejko Kruger, Yi‐Chun Kuan, Mitchell K. P. Lai, Karen Mather, Emily Meyers, Njideka Okubadejo, Stanley Onyango, Norfadilah Rajab, Maria Vania Rocha da Silva Nunes, Anna Rosenberg, Gessica Sala, Rosa Maria Salinas Contreras, Shahin Sayed, Margaret Scales, Seong Hye Choi, Hamid Sohrabi, Hilkka Soininen, Taiki Sugimoto, Ezequiel Surace, Yongxiang Wang, Henrik Zetterberg, Marissa Zwan, Ari Solomon, Francesca Mangialasche, Miia Kivipelto, the World‐Wide FINGERS network

**Affiliations:** ^1^ FINGERS Brain Health Institute Stockholm Sweden; ^2^ Division of Neurogeriatrics, Center for Alzheimer Research, Department of Neurobiology Care Sciences and Society, Karolinska Institutet Solna Stockholm Sweden; ^3^ Division of Clinical Geriatrics, Center for Alzheimer Research, Department of Neurobiology Care Sciences and Society, Karolinska Institutet Huddinge Stockholm Sweden; ^4^ Ageing Epidemiology Research Unit, School of Public Health Imperial College London London UK; ^5^ Department of Epidemiology and Prevention Wake Forest University School of Medicine Winston Salem North Carolina USA; ^6^ Brain and Mind Institute Aga Khan University Nairobi Kenya; ^7^ Brain Imaging Centre HUN‐REN Research Centre for Natural Sciences Budapest Hungary; ^8^ Department of Neurology Clínica Universidad de Navarra Pamplona Navarra Spain; ^9^ Centre for Age‐Related Medicine (SESAM) Stavanger University Hospital Stavanger Norway; ^10^ Behavioral and Cognitive Neurology Unit, Faculdade de Medicina Universidade Federal de Minas Gerais Belo Horizonte Brazil; ^11^ Center for Research and Memory Clinic CITA‐alzheimer Foundation Donostia‐San Sebastian Spain; ^12^ Barcelonaβeta Brain Research Center (BBRC) Pasqual Maragall Foundation Barcelona Spain; ^13^ Hospital del Mar Research Institute Barcelona Spain; ^14^ School of Medicine and Surgery and Milan Center for Neuroscience (NeuroMI) University of Milano‐Bicocca Milano Italy; ^15^ Fondazione IRCCS San Gerardo dei Tintori Monza Italy; ^16^ Deutsches Institut für Demenz Prävention (DIDP) Medical Faculty Saarland University Homburg Germany; ^17^ Department of Experimental Neurology Saarland University Homburg Germany; ^18^ UNSW Ageing Futures Institute University of New South Wales Sydney Australia; ^19^ School of Psychology University of New South Wales Sydney Australia; ^20^ Brain Health and Dementia Centre Neuroscience Research Australia Sydney Australia; ^21^ Department of Psychiatry, School of Medicine University of Pittsburgh Pittsburgh Pennsylvania USA; ^22^ Alzheimer's Disease Research Center University of Pittsburgh Pittsburgh Pennsylvania USA; ^23^ Unit for Brain Aging and Dementia, Department of Geriatric Medicine Dokuz Eylul University, School of Medicine Izmir Turkey; ^24^ Transversal Translational Medicine, Luxembourg Institute of Health (LIH) Strassen Luxembourg; ^25^ Luxembourg Research Clinic Centre Hospitalier de Luxembourg Luxembourg Luxembourg; ^26^ Translational Neuroscience, Luxembourg Centre for Systems Biomedicine (LCSB) University of Luxembourg Belvaux Luxembourg; ^27^ Department of Neurology, School of Medicine, College of Medicine Taipei Medical University Taipei City Taiwan; ^28^ Department of Pharmacology, Yong Loo Lin School of Medicine National University of Singapore Singapore Singapore; ^29^ Memory, Aging and Cognition Center National University Health Systems Singapore Singapore; ^30^ Centre for Healthy Brain Ageing, Discipline of Psychiatry and Mental Health, School of Clinical Medicine University of New South Wales Sydney Australia; ^31^ Alzheimer's Association Chicago Illinois USA; ^32^ Neurology Unit, Department of Medicine, Faculty of Clinical Sciences, College of Medicine University of Lagos Lagos, Lagos State Nigeria; ^33^ Centre for Healthy Aging and Wellness Faculty of Health Science Universiti Kebangsaan Malaysia Kuala Lumpur Malaysia; ^34^ Faculty of Health Sciences and Nursing, Centre for Interdisciplinary Research in Health Universidade Católica Portuguesa Lisbon Portugal; ^35^ Department of Public Health, Lifestyles and Living Environments Finnish Institute for Health and Welfare Helsinki Finland; ^36^ Institute of Clinical Medicine, Department of Neurology University of Eastern Finland Kuopio Finland; ^37^ National Institute of Neurology and Neurosurgery Manuel Velasco Suarez Mexico City México; ^38^ Department of Pathology Aga Khan University Hospital Nairobi Kenya; ^39^ Department of Neurology Inha University College of Medicine Incheon Republic of Korea; ^40^ Centre for Healthy Ageing, Health Futures Institute Murdoch University Perth Western Australia Australia; ^41^ School of Psychology, College of Health and Education Murdoch University Perth Western Australia Australia; ^42^ Alzheimer's Research Australia Perth Western Australia Australia; ^43^ Brain Research Unit University of Eastern Finland Kuopio Finland; ^44^ Department of Prevention and Care Science Research Institute National Center for Geriatrics and Gerontology Obu Japan; ^45^ Laboratorio de Enfermedades Neurodegenerativas, Instituto de Neurociencias (CONICET‐Fleni) Buenos Aires Argentina; ^46^ Department of Neurology Shandong Provincial Hospital Affiliated to Shandong First Medical University Jinan Shandong P.R. China; ^47^ Institute of Brain Science and Brain‐Inspired Research Shandong First Medical University and Shandong Academy of Medical Sciences Jinan Shandong P.R. China; ^48^ Department of Neurobiology, Care Sciences and Society Aging Research Center, Karolinska Institutet Stockholm Sweden; ^49^ Department of Psychiatry and Neurochemistry, Institute of Neuroscience and Physiology the Sahlgrenska Academy at the University of Gothenburg Mölndal Sweden; ^50^ Clinical Neurochemistry Laboratory Sahlgrenska University Hospital Mölndal Sweden; ^51^ Department of Pathology and Laboratory Medicine University of Wisconsin School of Medicine and Public Health Madison Wisconsin USA; ^52^ Wisconsin Alzheimer's Disease Research Center University of Wisconsin School of Medicine and Public Health, University of Wisconsin‐Madison Madison Wisconsin USA; ^53^ Department of Neurodegenerative Disease UCL Institute of Neurology London UK; ^54^ UK Dementia Research Institute at UCL London UK; ^55^ Centre for Brain Research Indian Institute of Science Bangalore India; ^56^ Alzheimer Center Amsterdam, Neurology Vrije Universiteit Amsterdam Amsterdam the Netherlands; ^57^ Amsterdam Neuroscience, Neurodegeneration Amsterdam the Netherlands; ^58^ School of Nursing Yale University Orange Connecticut USA

**Keywords:** dementia risk reduction, fluid biomarkers, Global biorepository, lifestyle‐based intervention, prevention, World‐Wide FINGERS

## Abstract

**INTRODUCTION:**

Dementia prevention research increasingly uses multimodal interventions. Harmonized biomarker practices are essential to enable comparable cross‐population analysis with initiatives such as the World‐Wide FINGERS (WW‐FINGERS) network contributing to this effort.

**METHODS:**

To advance globally representative biomarker research in dementia prevention trials, we conducted a cross‐sectional, network‐wide survey of WW‐FINGERS teams assessing biospecimen collection, processing and storage, biomarker measurements, and readiness for harmonized procedures.

**RESULTS:**

Most studies (30/39) had or were developing biorepositories; four planned collection and five had none. Practices were broadly aligned, but remaining variability highlights priority targets for harmonization. Clinical biomarkers (lipids, glucose, liver/kidney function) were obtained even in lower‐resourced settings. Many studies measured Alzheimer's disease and related dementias (ADRD) biomarkers (blood‐based amyloid/tau and neurodegeneration; cerebrospinal fluid where feasible) and increasingly used multi‐omics platforms. Evidence‐based preanalytical guidance were provided.

**CONCLUSION:**

The WW‐FINGERS biorepository provides a world‐first platform for harmonized biomarker discovery across diverse populations in dementia prevention, helping to reduce inequities in research participation, accelerating globally representative strategies.

## Highlights

- WW‐FINGERS connects global teams for cohesive dementia risk reduction strategies
- A survey was launched mapping current biorepositories across WW‐FINGERS trials
- Results show a unique potential for future, large‐scale joint analyses
- The summarized findings aim to support prospective harmonization
- A unified biorepository aims to enhance global dementia risk reduction efforts

## 1 BACKGROUND

Dementia is a global public health challenge, given the rapid growth of affected individuals and lack of widely available effective therapeutics.^1^, ^2^ Interventions in at‐risk stages are believed to be efficient in slowing the onset and progression of neuropathological processes and cognitive symptoms.^3^, ^4^ Estimates suggest that 45% of global dementia cases are attributable to 14 potentially modifiable factors, including lifestyle, cardiometabolic, and/or environmental factors.^5^, ^6^ Consistently, the updated World Health Organization (WHO) guidelines, refer to 18 modifiable risk factors, and support multidomain approaches targeting several disease mechanisms simultaneously.^7^

The Finnish Geriatric Intervention Study to Prevent Cognitive Impairment and Disability (FINGER) was the first large‐scale multidomain randomized controlled trial (RCT) showing cognitive benefits in older adults at risk for dementia.^8^ The 2‐year FINGER intervention included nutritional advice, exercise, cognitive training, social activities, and management of cardiometabolic factors. This paradigmatic model is currently adapted globally in the World‐Wide FINGERS network (WW‐FINGERS, 70+ countries),^9^ representing the largest collaborative effort toward dementia risk reduction and prevention. Recently WW‐FINGERS RCTs further validated feasibility and efficacy of the multidomain approach across diverse populations and settings including: U.S. Study to Protect Brain Health Through Lifestyle Intervention to Reduce Risk (U.S. POINTER), Japan‐Multimodal Intervention Trial for the Prevention of Dementia (J‐MINT), Australian Maintain Your Brain (MYB), South Korean study to prevent cognitive impairment and protect brain health through lifestyle intervention in at‐risk elderly people (SUPERBRAIN), Malaysia Multidomain Intervention for Reversal of Cognitive Frailty (AGELESS), the SINgapore GERiatric Intervention Study to Reduce Cognitive Decline and Physical Frailty (SINGER) along LatAm‐FINGERS across Latin America.^10^, ^11^, ^12^, ^13^, ^14^, ^15^ The ongoing Africa‐FINGERS project further enriches cultural and genetic diversity.^16^

**RESEARCH IN CONTEXT**

1. **Systematic review**: The authors reviewed existing literature using traditional sources. A central gap is the absence of harmonized, scalable biorepository protocols for fluid biomarker collection, processing, storage, and analysis across dementia risk‐reduction trials. This limits cross‐study comparability, biomarker validation, and future pooled analyses. Limited evidence on molecular pathways underlying lifestyle intervention effects further underscores the need for coordinated biospecimen infrastructure to optimize intervention benefit.
2. **Interpretation**: The study presents findings from a survey carried out within the WW‐FINGERS framework by the Biorepository and Biomarkers International Working Group (WWF‐BB‐IWG). Results outline the status of WW‐FINGERS studies, identifying existing biorepositories, detailing pre/post analytic procedures and biomarker analyses. This manuscript serves as a foundation for ongoing and future collaborative analyses and projects initiated by the WWF‐BB‐IWG.
3. **Future directions**: By leveraging diverse population datasets, the next phase of the WWF‐BB‐IWG establishes an evidence‐based globally connected precision‐prevention framework. This will enable future joint analysis and support the identification of subgroup‐specific intervention responders accelerating scalable dementia prevention.

Additionally, innovative RCTs within the network are testing combinations of lifestyle interventions with nutraceuticals^17^, ^18^, ^19^, ^20^ or putative disease‐modifying treatments (DMTs, e.g., the MET‐FINGER trial)^21^ with encouraging results in feasibility and efficacy.

The WW‐FINGERS network activities organized by the Global Scientific Coordination Center at FINGERS Brain Health Institute and Alzheimer's Association have established the WW‐FINGERS International Working Groups (WWF‐IWGs). Each WWF‐IWG gathers network members with relevant expertise for the topic in focus (Figure 1), ensuring global representation and facilitating sharing of knowledge and expertise. The WWF‐IWGs have implemented and provide prospective methodological harmonizations to the network and broader scientific community, with attention to sustainability and inter‐ and intra‐regional disparities. Key priorities and characteristics are presented in Figure 1.

**FIGURE 1:**
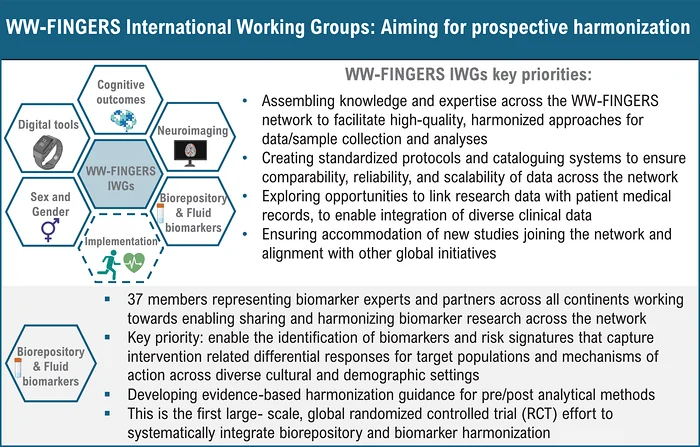
Overview of WW‐FINGERS IWG; Key priorities of the IWGs and main characteristics and scope of the WWF‐IWG on Biorepository and Fluid Biomarkers. IWG, International Working Groups; WW‐FINGERS, World‐Wide FINGERS.

Fluid biomarkers are increasingly used in the diagnostic criteria for Alzheimer's disease and related dementias (ADRD),^22^ reflecting diverse neuropathological processes.^23^, ^24^ In parallel, ‘omics‘‐approaches allow deep molecular profiling^25^, ^26^, ^27^, ^28^, ^29^, ^30^ identifying complementary biomarkers.^31^ Shifting from determining AD neuropathology by positron emission tomography (PET)‐neuroimaging or CSF to more accessible biospecimens, including blood or dried blood spots,^32^, ^33^, ^34^ can reduce costs and improve inclusion of under‐represented cohorts from low‐resource settings. Advances in analytical sensitivity and specificity have established blood‐based biomarkers (e.g., p‐tau217) among the most promising tools for reliably detecting AD pathology.^35^, ^36^ While pharmacological trials often use fluid biomarkers to assess eligibility, monitor target engagement, safety, and response,^37^ lifestyle‐based intervention studies were historically less likely to establish biorepositories, typically measuring biomarkers in sub‐samples for exploratory analysis, (e.g., intervention mechanisms).^38^ The integration of less invasive sampling methods (e.g., blood) could facilitate broader biomarker use in prevention trials supporting deeper phenotyping and enabling optimization of RCTs design (e.g., participants allocation to treatment arms), while maximizing trial efficacy.

The establishment of the WWF‐IWG on Biorepository and Fluid Biomarkers (WWF‐BB‐IWG) aims to enable identification of differential risk profiles/prevention potential phenotypes, and measures linked to interventions’ responses across diverse demographic and cultural settings. Developing a unified harmonization approach for various biofluids will reduce pre‐ and /post‐analytical variability, improve cross‐study comparability,^39^ and support global analytical testing, using validated assays across different vendors.

This article presents the findings of a global survey and proposes guidance for prospective harmonization to establish standardized methodologies and joint analyses for the lifestyle‐based dementia prevention field. Beyond supporting harmonization within WW‐FINGERS, this work provides a practical framework for the wider dementia prevention research community for collaborative and secondary analyses across diverse populations.

## 2 METHODS

### 2.1 WWF‐BB‐IWG survey

We built a cross‐sectional survey, which was distributed digitally via the Research Electronic Data Capture tool (REDCap,^40^ version 14.0.5) hosted at Karolinska Institutet, Stockholm, Sweden. The survey questions were tailored for three study phases commonly represented in the WW‐FINGERS network: planning, ongoing, and completed, and consisted of multiple‐choice or open‐ ended questions (Supporting Information 1) organized in four parts. The first section collected general information regarding the studies (e.g., name, country, status, cohort key characteristics, links to existing biorepository). The second part focused on information about fluid sample collection routines, main characteristics of sample handling, and storage practices and frequency. The third part addressed types of biomarkers analyses, from well‐established clinical markers to AD biomarkers, along with exploratory analyses such as proteomics, metabolomics, epigenomics, lipidomics, or genome‐wide association studies (GWAS). Finally, respondents could provide additional information on other biomarkers, planned analyses, and preferred analytical platforms. No personal data/information were collected.

### 2.2 Data collection

A secure link to the survey was sent out by email to the WW‐FINGERS network, with additional invitations to complete the survey sent out to new countries/study sites joining the network. Participation in the survey was optional. The last invitation to participate and update the study status was sent out in August 2025. The survey analyses presented in this paper include survey responses received by September 2025. Trial status (e.g., ongoing or completed, Table 1) was updated as of September 2026.

**TABLE 1 alz71949-tbl-0001:** Key characteristics of the survey respondent World‐Wide‐FINGERS Network Studies^a^

Study name	Study site country	Cohort size	Source of participants	Population characteristics	Linked biorepository	Sample type
Africa‐FINGERS^16^	Nigeria	500‐1000	General population/memory clinic	At‐risk	Planning	plasma, serum whole blood
	Kenya				Yes	
AGELESS^41^	Malaysia	100‐500	Other (community dwelling)	At‐risk	Planning for follow‐up	plasma, serum, whole blood
AMETHYST^c^	Slovakia	< 100	General population/memory clinic	At‐risk/MCI	Yes	plasma, serum, CSF, stool
AU‐ARROW^42^	Australia	500‐1000	General population	At‐risk	Yes	plasma, serum, whole blood, urine, platelets
CADAS^43^, ^c^	Dominican Republic	> 1000	General population	Elderly	Yes	whole blood, plasma, serum
	Puerto Rico					
	Cuba					
Can THumbs UP^44^	Canada	100‐500	General population	At‐risk/MCI	Yes	saliva
CINGER	Cyprus	100‐500	Memory clinic	Healthy/MCI/AD/dementia	Planning	plasma, serum, whole blood, platelets
CITA GO‐ON^45^	Spain	>1000	General population	At‐risk/ MCI	Yes	plasma, serum, whole blood, platelets
COGNET^c^	Hungary	100‐500	General population/ memory clinic		Yes	not specified
Cuban Aging and Alzheimer´s Study^46^, ^c^	Cuba	>1000	General population		Planning	not specified
DemenPrev	Portugal	<100	Community dwelling	At‐risk	No	
eFINGER PRINT^47^	UK	100‐500	General population	Healthy	Yes	saliva
EMBRACE^c^	Slovakia	<100	General population	At‐risk/ MCI	Yes	plasma, serum CSF
FINGER^8^	Finland	>1000	General population	At‐risk	Yes	plasma, serum, whole blood
GenWellBrave	Australia	<100	General population	Healthy	No	
GERICO^c^	Italy	>1000	Memory clinic	Healthy/at‐risk/MCI/AD/dementia	Yes	plasma, serum, whole blood
GINGER^48^	Greece	<100	Memory clinic	At‐risk	Planning for follow‐up	not specified
HUN‐MLI‐BRAIN	Hungary	<100	General population	Healthy/at‐risk	Yes	plasma
ICMR NIN^c^	India	500‐1000	General population	Healthy/at‐risk	Yes	plasma, serum, whole blood
INCLUSIVE	Australia	<100	Other	MCI	No	
INTEGRITY	Australia	100‐500	General population	Healthy	No	
IN‐TeMPO^49^	Italy	>1000	General population	At‐risk	Yes	plasma, whole blood, urine, PBMC
Izmir‐FINGER	Turkey	100‐500	Memory clinic	AD/dementia	Planning	not specified
J‐MINT^11^	Japan	500‐1000	General population/memory clinic	MCI	Yes	plasma, serum
LatAm‐FINGERS^50^	Argentina	> 1000^b^	General population/memory clinic	At‐risk	Yes	plasma, serum
	Bolivia				Yes	plasma, serum
	Brazil				Yes	plasma, serum
	Chile				Yes	plasma, serum
	Colombia				Yes	plasma, serum, DNA
	Costa Rica				Yes	plasma, whole blood
	Dominican Republic				Yes	plasma, serum, whole blood
	Ecuador				Yes	plasma, serum
	Mexico				Yes	plasma, serum
	Peru				Yes	plasma, serum, whole blood
	Puerto Rico				Yes	plasma, serum
	Uruguay				Yes	plasma, serum
LETHE^51^	Austria	100‐500	General population/memory clinic			plasma, serum
	Finland			At‐risk	Yes	
	Italy					
	Sweden					
LIBRA‐Net^c^	Slovakia	< 100	General population/memory clinic	At‐risk/MCI	Yes	plasma, serum, CSF
MET‐FINGER^21^	UK	500‐1000	General population	At‐risk	Yes	
	Finland					plasma, serum, whole blood
	UK					
MIND‐ADmini^19^, ^52^	Finland		General population/memory clinic	MCI (prodromal AD)	Yes	
	France	<100				plasma, serum, whole blood
	Germany					
	Sweden					
MIND‐China^53^	China	>1000	General population	Elderly	Yes	plasma, serum, whole blood
MYB^13^, ^54^	Australia	>1000	General population	At‐risk	Yes	whole blood, saliva
NL‐FINGER^18^	Netherlands	>1000	General population	At‐risk	Yes	plasma, serum
PENSA^55^	Spain	100‐500	General population	At‐risk	Yes	plasma, urine, saliva, stool
SINGER^56^	Singapore	> 1000	General population/memory clinic	At‐risk	Yes	plasma, serum, whole blood
SUPERBRAIN^14^, ^57^	South Korea	100‐500	Memory clinic	At‐risk/ MCI	Yes	plasma, serum
SWISS‐FINGER	Switzerland	100‐500	General population/memory clinic	Healthy/at‐risk/MCI/ AD/dementia	Yes	plasma, serum, CSF
SYNERGIC 2^58^	Canada	500‐1000	Other	MCI	Yes	saliva
TIGER^59^	Taiwan	100‐500	General population	Healthy (comorbidities)	Yes	serum
U.S.‐POINTER^60^	USA	>1000	General population	At‐risk	Yes	plasma, whole blood

*Note*: At‐risk population represents people who have objectively measured normal cognition with evidence of risk factors, presence of subjective cognitive decline, or memory complaints (no dementia).

Abbreviations: AD, Alzheimer's disease; CSF, cerebrospinal fluid; MCI, mild cognitive impairment; PBMC, peripheral blood mononuclear cell; RCT, randomized controlled trial; WW‐FINGERS, World‐Wide FINGERS.

^a^ The table includes both multidomain trials and observational studies with RCT‐readiness potential conducted by responding WW‐FINGERS network members. Completed studies are denoted by light grey background color, while ongoing studies have white background color.

^b^ Denotes cohort size for the entire study, with an equal number of participants planned to be recruited across the participating countries.

^c^ Denotes observational studies with readiness potential used for screening and/or recruitment to WW‐FINGERS RCT.

### 2.3 Survey data analysis

To ensure accuracy and consistency, REDCap survey data were extracted and analyzed independently by three people (V.A., M.D., and E.B.). The dataset was examined for missing data at a case level. Missingness ranged from 0% to 30%, with most of the categories having < 10% missing information. Any discrepancies were discussed and resolved through consensus and by asking for clarification from relevant teams to ensure data integrity. Analyses were further performed in Microsoft Excel and GraphPad Prism (version 10.1.2). Descriptive statistics were used to assess the data. Figures were prepared using BioRender, or Graphpad Prism.

## 3 RESULTS

### 3.1 Survey respondents

The survey on biorepositories and biomarkers was distributed across 76 WW‐FINGERS member teams and was completed by 48[Table alz71949-tbl-0001] respondents (66%), reflecting broad engagement across the network. Together, it represents 39 different WW‐FINGERS studies, including both interventional randomized controlled trials (RCTs) (*N* = 31) along with observational studies (*N* = 8) with readiness cohort potential, supporting the development of a biorepository for fluid biomarkers (Table 1). Some of the trials are conducted across multiple countries but with one joint biorepository (e.g., LETHE, MIND‐AD, MET‐FINGER), whereas other trials have separate biorepositories for each country (e.g., Lat‐Am FINGERS, Africa‐FINGERS) and thus multiple survey respondents. Survey responses were received from respondents representing completed (*N* = 18) and ongoing (*N* = 21) studies (Table 1).

### 3.2 Study populations

Participant populations and sample sizes vary across WW‐FINGERS studies, reflecting differences in recruitment setting, dementia‐risk profile and cognitive status rather than fundamentally different prevention objectives (all included multidomain lifestyle‐based approaches for dementia risk reduction). The typical sample size for RCTs varies between 101 and 500 participants (*N* = 11), with six studies enrolling a population of fewer than 100 participants, five having a population between 501 and 1000 participants, and nine reporting a study population greater than 1000 participants (Table 1). While most RCTs recruited from the general population (*N* = 16), seven RCTs also enrolled participants from clinical settings, such as memory clinics (Table 1). Four studies enrolled participants exclusively from memory clinics, and two recruited from other sources (Table 1). The study participant group exhibits some heterogeneity, covering the at‐risk continuum for ADRD. Among the reported RCTs, the cohorts mainly included populations at risk of dementia (*N* = 15 intervention trials) defined as individuals with objectively measured normal cognition with evidence of risk factors, presence of subjective cognitive decline, or memory complaints (no dementia). Other studies have included either healthy participants with modifiable risk (*N* = 4) or participants with memory impairment, such as mild cognitive impairment (MCI) (*N* = 4) (Table 1). Often, the study population comprised individuals across two categories (*N* = 9) or across the whole ADRD continuum (*N* = 2). Similar features, in terms of sample size, setting, and population characteristics, are present for observational cohorts with readiness potential (Table 1). This population heterogeneity provides an opportunity for future harmonized analyses examining the generalizability and modifiers of biomarker profiles and intervention response across different risk groups and settings.

### 3.3 Biorepositories

Whereas biomarkers provide specific measurable indicators at a given time, biorepositories offer a long‐term resource by preserving high‐quality samples for upcoming analyses. Therefore, establishing robust biorepositories is essential for facilitating the identification of novel biomarkers, the validation of emerging assays, joint analysis, and reanalysis using technologies that go far beyond the initial measurement plan. Almost all teams (*N* = 41), collected biosamples, or are planning to (*N* = 6), including 27 RCTs. Most of the surveyed studies collect plasma (79%) and serum (74%) from the study participants (Figure 2A, Table 1). Almost half of them also collect whole blood samples (47%). Other fluid samples, such as CSF (10%), RNA collecting tubes (8%), urine (8%), and saliva (10%), are also collected by some respondents. Further biospecimens, such as stools, and hair, are less frequently collected (*N* = 3). Planned biorepository storage time varies across studies, with 13% of the respondents having a storage time less than 5 years, 24% between 5 ‐and 10 years, 26% between 10‐ and 15 years, and finally 32% of respondents storing samples for over 15 years (Figure 2B). In general, most countries from the Global South have shorter storage time, highlighting the need for sustainable solutions. The samples are kept mainly at –80°C (71%), but in some studies, samples are stored at both ‐80°C and ‐20°C (16%) and rarely at ‐20°C only (8%) (Figure 2C). One RCT reported sample storage in liquid nitrogen. In most cases, samples were stored at each study's own facilities, and only a few reported uses of an outsourced biobank (Figure 2D).

**FIGURE 2:**
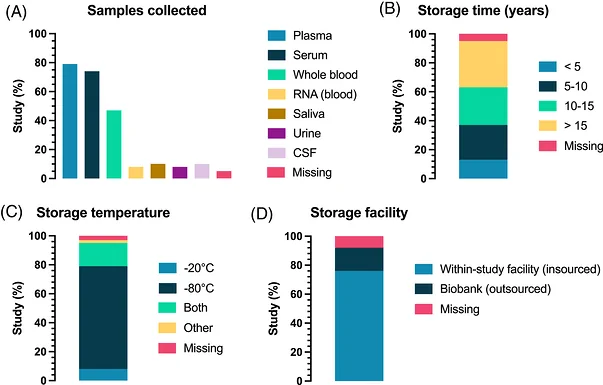
Sample Collection and Storage of the World‐Wide‐FINGERS Biorepository. (A) Different biofluid samples collected among survey respondents. (B) Planned biorepository storage times, (C) temperatures, and (D) facilities for the collected samples.

Of respondents who had collection of blood samples (either as plasma, serum, and/or whole blood), around 60% collected samples for the entire cohort, including 22 RCTs. Excluding responses with missing information (10%), all remaining respondents collected plasma in ethylenediaminetetraacetic acid (EDTA) tubes. Of these, 76% used EDTA exclusively, 11% used both EDTA and heparin, and 3% collected plasma using EDTA, heparin, and citrate tubes (Figure 3A). The majority (83%) of responding studies reported sample collection in fasting conditions and during the morning (81%) (Figure 3B‐C). The time interval between consecutive sampling was up to 6 months in 28% of respondents, between 6‐ and 12 months in 33%, or > 12 months in 25% of the respondents. (Figure 3D). In cases when cognitive testing and/or brain imaging were performed, they mostly occurred within 0‐6 months of sample collection (Figure 3E). Three out of the four studies collecting CSF reported sampling in subgroups at intervals of 6‐12 months, and one reported sample collection after more than 12 months (data not shown). Two out of the three respondents collecting urine performed the collection in the entire cohort every six months, while the remaining had an 18‐ month interval (data not shown). Similarly, 80% of the responding studies that were collecting saliva samples performed it across the entire cohort, and 20% only collected saliva for DNA extraction from participants who did not consent to donate a blood sample. Saliva collection occurred at the start and end of the trial in 40% of responses or at baseline only in another 40% of responses. In most of them, no specific time of day for saliva collection was reported (data not shown).

**FIGURE 3:**
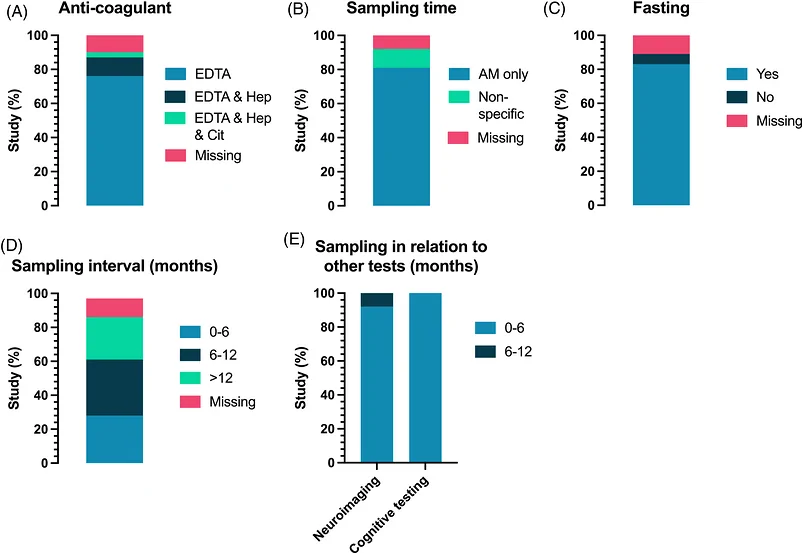
Preanalytical handling of blood samples among responding World‐Wide FINGERS partners. (A) The choice of anti‐coagulant for plasma collection. (B) Time and (C) fasting status of study participants for blood sampling. (D) Interval of consecutive blood sampling. (E) The timeframe for blood sampling in relation to neuroimaging and cognitive testing. EDTA, ethylenediaminetetraacetic acid; Hep, heparin; Cit, citrate.

### 3.4 Biomarkers

Studies collecting biosamples, including 15 RCTs, have or are currently measuring ADRD‐related CSF and/or plasma biomarkers. These include 37% measuring Aβ peptides, 30% total tau, 42% phosphorylated tau species (p‐tau), 37% neurofilament light (NfL), and 33% glial fibrillary acidic protein (GFAP), with 30% of the studies including all the above biomarkers (Figure 4A). Apolipoprotein E (*APOE*) genotyping is currently performed or planned to be performed in 74% of studies (Figure 4B) including 18 RCTs providing an important foundation for potential future pooled or joint analyses. In addition, although only nine RCTs have reported thus far further molecular phenotyping across various platforms, several studies intend to integrate approaches such as proteomics, metabolomics, epigenomics, lipidomics, and/or GWAS genome‐wide association study (Figure 4B) contingent on the availability of funding.

**FIGURE 4:**
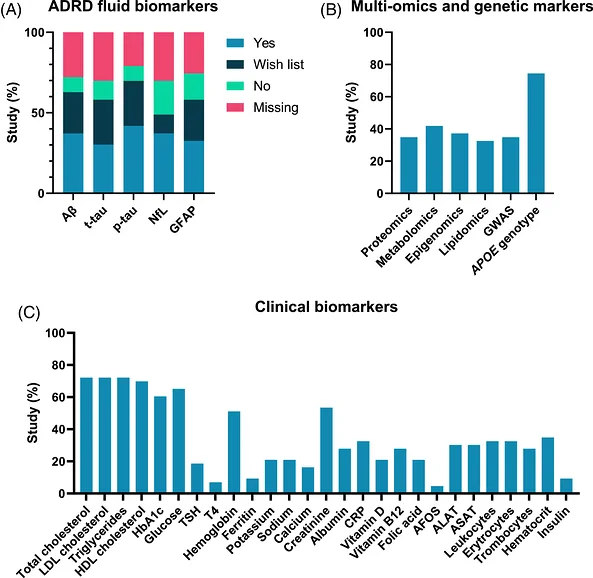
Biomarkers analyzed across World‐Wide‐FINGERS studies. (A) Studies measuring AD biomarkers; have measured (yes), wish to measure (wish list), or not planning to measure (no). (B) Studies currently performing or planning to perform exploratory ‐omics biomarker studies. (C) Studies measuring other clinically relevant biomarkers. Aβ, amyloid beta; AD, Alzheimer's disease; AFOS, alkaline phosphatase; ALAT, alanine aminotransferase; ASAT, aspartate aminotransferase; CRP, C‐reactive protein; GFAP, glial fibrillary acidic protein; GWAS, genome‐wide association study; HbA1c, hemoglobin A1c; HDL, high‐density lipoprotein; LDL, low‐density lipoprotein; NfL, neurofilament light; p‐tau, phosphorylated tau; T4, thyroxine; TSH, thyroid‐ stimulating hormone; t‐tau, total tau.

The majority of studies (72%) collected blood samples for validated, routine clinical laboratory biomarkers commonly used as indicators for cardiometabolic risk factors, which are also used to monitor intervention safety and effects (Figure 4C). The most measured markers across studies include total cholesterol, LDL and HDL cholesterol, triglycerides, and glucose or HbA1c. Creatinine, and hemoglobin were also frequently assessed, though to a lesser extent.

## 4 DISCUSSION

A strategic priority for advancing dementia prevention research is the early identification of individuals at‐risk before disease onset, which requires robust, well‐characterized, and adequately powered datasets. While individual prevention trials generate invaluable longitudinal data, their sample sizes often limit statistical power for biomarker discovery, validation, and subgroup analyses. Establishing a coordinated global biorepository, such as within the WW‐FINGERS, enables harmonized and scalable biomarker research. Toward this, the WWF‐BB‐IWG has surveyed the status of the cross‐network biorepository providing a platform for biomarker discovery across diverse geographies, serving as a unique and transformative resource for the whole dementia prevention research community.

This effort highlighted both the constraints, such as limited per‐trial sample, variability in collection protocols, and the considerable opportunity for joint analyses (e.g. obaseline characteristics, intervention benefit). This coordinated biorepository framework, accelerated by emerging linked initiatives such as the global neurodegeneration proteomics consortium,^26^ allows cross‐trial validation of biomarkers and facilitates analyses of intervention‐response. Simultaneously, careful reflection is required regarding methodological heterogeneity across RCTs, including target population, biospecimen collection, processing pipelines, storage conditions, and assay platforms.

### 4.1 Fluid biomarkers in multidomain lifestyle‐based interventions

Biomarkers play an essential role in multidomain lifestyle intervention trials, serving as eligibility and outcome measures along exploratory tools of mechanisms of resilience or susceptibility (Table 2). Clinically validated biomarkers‐ particularly cardiometabolic measures such as blood lipids, glucose, HbA1c, blood pressure, and inflammatory markers‐ have been widely used in prevention RCTs,^10^, ^21^ providing actionable endpoints linking lifestyle modification (e.g., diet, physical activity, vascular risk management) to changes in dementia risk. Although not yet uniformly available across all studies, emerging blood biomarkers such as Aβ, p‐tau, and NfL may capture biological responses to interventions, anchoring lifestyle‐based intervention outcomes to AD pathophysiology.^61^, ^62^ However, it is important to recognize that these biomarkers remain largely exploratory in the context of lifestyle‐based trials and have not yet been sufficiently validated as surrogate endpoints capable of supporting regulatory decisions or serving as primary clinical trial outcomes in multidomain lifestyle interventions.

**TABLE 2:** Biomarker protocols and sample types for outcome and exploratory measures in multidomain lifestyle dementia intervention trials

**Biorepository** ^63^	Describes the main elements of a biorepository: governance, standards, best practice operational workflows and specimen handling procedures	
**Blood** ^39^, ^64^, ^65^	Practical instructions including study design, pre‐analytical handling, sample collection, storage and AD related biomarker measurements	
**CSF** ^66^, ^67^	Protocols for cerebrospinal fluid collection and storage, preanalytical factors, routine analyses of AD biomarkers	
**Other biosamples** ^68^, ^69^, ^70^, ^71^	Current status of dementia biomarkers in other biosamples (saliva, urine, tears, microbiome)	
Fluid biomarker category	Examples	Purpose
**Clinical parameters**	Blood glucose, HbA1c, insulin resistance indices, blood lipids (total cholesterol, LDL, HDL, triglycerides), inflammatory markers (CRP, IL‐6)	Monitor vascular and metabolic health; primary outcomes or intermediate endpoints linking lifestyle change to dementia risk reduction.
**Safety biomarkers**	Liver enzymes (ALT, AST), renal function (creatinine, eGFR), hematology panels	Ensure safety and tolerability of interventions (diet, exercise, pharmacological or nutraceutical components).
**Pharmacodynamic /pharmacokinetic markers**	Drug/nutrient metabolites fitamin D, omega‐3 fatty acid levels, homocysteine	Assess adherence, intervention exposure, and biological activity of nutritional or pharmacologic components.
**Genetic biomarkers**	APOE genotype; polygenic risk scores (PRS) for AD and vascular risk	Evaluate genetic risk and explore moderation of intervention effects.
**Biomarkers of neurodegeneration and dementia‐related mechanisms**	Plasma/CSF Aβ42/40, p‐tau181, p‐tau217, NfL, GFAP, ‐omics	Outcome or exploratory measures linking interventions to AD pathophysiology; limited availability across geographies due to cost/infrastructure.

Abbreviations: AD, Alzheimer's disease; APOE, apolipoprotein E; CRP, C‐reactive protein; CSF, cerebrospinal fluid; eGFR, estimated glomerular filtration rate; GFAP, glial fibrillary acidic protein; HDL, high‐density lipoprotein; IL, interleukin; LDL, low‐density lipoprotein; NfL, neurofilament light chain;

Multiple biological processes, such as inflammation, glucose/lipid metabolism, autophagy, protein aggregation, oxidative stress, and synaptic plasticity, have been previously linked to lifestyle factors.^5^, ^38^ By detecting subtle molecular changes before cognitive symptoms emerge, these biomarkers can enhance early intervention strategies and improve our understanding of how modifiable lifestyle factors influence neurodegeneration and brain resilience. In addition, safety and, where applicable, pharmacodynamic/pharmacokinetic markers also support monitoring and mechanistic understanding in RCTs combining non‐pharmacological and pharmacological components.

Ultimately, integrating fluid biomarkers into multidomain trials strengthens their potential to generate personalized approaches for AD prevention and treatment.^38^

### 4.2 Target populations in WW‐FINGERS and other prevention trials

In prevention trials, identifying individuals who would benefit most from lifestyle‐based intervention has relied heavily on risk scores, which are based on non‐modifiable and modifiable risk factors (e.g., CAIDE, LIBRA, WHICAP, ANU‐ADRI, CogDrisk).^72^, ^73^, ^74^, ^75^ Integrating a clinical‐biological framework into eligibility criteria for lifestyle‐based interventions may further improve precision and clinical effectiveness. This combined clinical‐biological eligibility model is currently being applied in the WW‐FINGERS network in the MET‐FINGER trial, where lifestyle intervention combined with administration of metformin is tested in a population at‐risk of dementia with pre‐diabetes indications.^21^ In other complex chronic disorders such as atrial fibrillation, treatment strategies commonly involve a combination of drugs adapted to individual profiles.^76^ A similar approach can be used in dementia, where WW‐FINGERS may serve as a platform for identifying molecular mediators of cognitive change matched to multi‐dimensional risk profiles.

Evidence from several prevention trials also suggests that genetic risk may modify response to lifestyle interventions. Intervention efficiency among *APOE ε4* carriers has been reported in FINGER RCT,^77^ SUPERBRAIN‐MEET,^78^ J‐MINT,^11^ MAPT^79^ or U.S. POINTER^10^ trials. A recent meta‐analysis showed a significantly higher effect in *APOE ε4* carriers compared to non‐carriers.^80^ Other studies^81^, ^82^ have extended this observation, examining polygenic risk scores as potential modifiers of intervention response,^82^ underscoring the importance of incorporating genetic risk profiling into biomarker frameworks to identify subgroups who may respond differentially to interventions. Biological markers may also help identify individuals who derive greater benefit from lifestyle interventions, e.g. in the FINGER RCT, participants with a higher serum level of the hypercholesterolemia‐related 27‐hydroxycholesterol benefitted more from the intervention.^83^ In addition, leukocyte telomere length maintenance was reported to be associated with more pronounced cognitive intervention benefits.^84^ Moreover, subgroup analyses from J‐MINT indicated that participants with uncontrolled vascular risk factors (e.g., high blood pressure, HbA1c, or dyslipidemia) or participants with higher plasma GFAP concentrations‐reflecting astroglial activation‐derived greater benefit from the intervention.^85^

### 4.3 Developing a WW‐FINGERS biorepository, a platform for the development of future prevention trials

The overarching aim of the WW‐FINGERS network is to increase knowledge about dementia and multidomain lifestyle‐based intervention among diverse populations.^9^ Because participating cohorts differ demographically, socio‐economically, clinically, and ethnically/genetically, harmonizing technical and methodological procedures is essential. To support this, the WWF‐BB‐IWG, mapped the current pre‐analytical practices, storage protocols, and biomarker assessments to generate general guidance for prospective harmonization (Figure 5, Table 2) to facilitate future joint analysis using harmonized collection and processing protocols.

**FIGURE 5:**
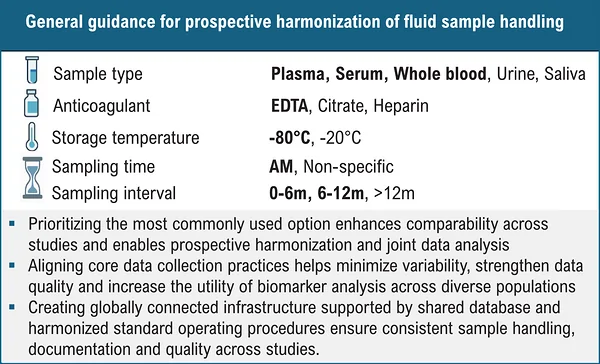
General guidance for prospective harmonization of fluid sample handling in dementia prevention trials.

Survey responses highlighted considerable convergence across key biorepository practices, with existing variability underscoring the need for standardized, sustainable procedures to guide future WW‐FINGERS harmonization and accommodate diverse setting. We further provide a flexible framework that integrates existing best practices and standard operating procedures (SOPs, Table 2)‐ aligned to previous biomarker harmonization efforts such as the Alzheimer's Association Global Biomarker Standardization Consortium (GBSC)^86^ for sampling methods and storage conditions. Individual study sites can adapt these, aligning with their capacity and resources.

Despite these important insights, findings are subject to inherent methodological and practical limitations. Data reported were only collected within existing members of the WW‐FINGERS network, limiting generalizability. Also, responses were not mandatory, they were self‐reported and may therefore be subject to reporting bias and variable interpretation of terminology across sites. Furthermore, several network members, mostly with trials in the planning phase, have not completed the survey. While this may limit full representativeness, major studies across continents‐ including low‐, middle‐, and high‐income countries, are nonetheless well captured. In addition, some items had missing data, and variations in local practices or definitions may have introduced inconsistencies in how questions were answered. These factors should be considered when interpreting these findings.

### 4.4 Biomarker accessibility: Challenges and opportunities across borders

The use and accessibility of biomarkers vary significantly across geographies, reflecting differences in infrastructure, healthcare systems, and resource availability. For example, while large‐scale biomarker analyses, including genetics, plasma p‐tau, and other neurodegeneration markers, have been conducted in high‐resource settings, many low‐ and or middle‐income country studies might face challenges in biomarker analysis and/or long‐term sample storage. Barriers include high costs of assays, cold‐chain requirement for specimen preservation, limited availability of biobanking infrastructure, and variable regulatory environments.

These constraints can significantly limit the systematic integration of biomarker endpoints in global dementia prevention trials. Addressing these disparities, therefore, has become a priority for international collaborative initiatives. Efforts such as our WWF‐BB‐IWG seek to harmonize protocols for biospecimen collection, processing, storage, and analytical pipelines while considering differences in scientific rigor, local infrastructure, and feasibility.

Emerging[Fig alz71949-fig-0006] technologies are enhancing equitable biomarker integration in global studies. Minimally invasive methods like dried blood spot (DBS) collection, capillary microsampling, and saliva assays are alternatives to traditional blood collection, reducing costs and simplifying logistics. DBS‐based proteomic and genomic assays, for example, have shown promise for measuring inflammatory markers, metabolic biomarkers, and certain neurodegeneration‐related proteins,^87^, ^88^, ^89^ while saliva offers a scalable platform for genomic, epigenomic, hormonal, and emerging proteomic analyses relevant to neurodegeneration and brain health.^68^ Advances in ultra‐sensitive assays further enable these approaches for large‐scale studies and decentralized trials. Expanding the use of accessible biosampling technologies alongside traditional laboratory‐based approaches will be critical for democratizing access to robust biophenotyping and dementia biomarker research.

## 5 CONCLUSION AND NEXT STEPS

Harnessing rich datasets from highly diverse populations, lays the foundation for globally scalable, tailored, evidence‐based prevention strategies that accelerate dementia prevention trials. Such global harmonization of data collection, processing, and analyses, requires a deep understanding of target populations, including diverse sociodemographic and economic contexts. Beyond enabling cross‐country comparisons, the integration of biorepository datasets will unlock opportunities to study dementia prevention in association with rare conditions, genetic variants, or other sub‐populations that individual RCTs alone may lack the power to address, paving the way for globally relevant prevention strategies.

We aim to establish a globally connected infrastructure‐ anchored by a shared database and harmonized SOPs‐ to ensure consistent sample handling, documentation, and high quality across all studies (Figure 6). This unified framework will facilitate potential future joint analyses and cross‐trial subgroup discoveries, particularly for identifying best responders within intervention groups. Moreover, several studies are also planning longitudinal follow‐up, where biomarkers may help monitor treatment response, maintenance of effects, and related outcomes over time. Importantly, this mapping exercise has practical utility beyond describing current WW‐FINGERS network activity. It identifies procedures already sufficiently convergent to support prospective harmonization, highlights pre‐analytical sources of variability requiring standardization, and provides a framework for investigators developing biomarker‐enabled dementia prevention studies.

**FIGURE 6 alz71949-fig-0006:**
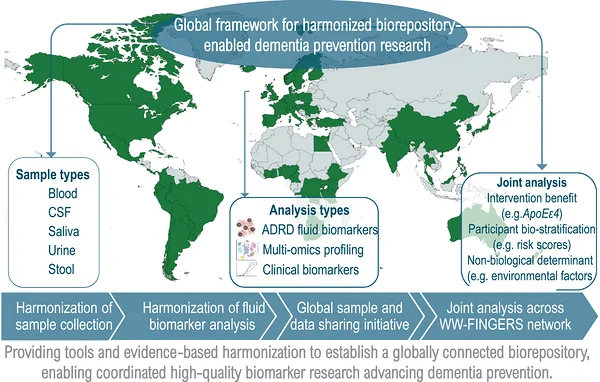
Global framework for harmonized biorepository‐enabled dementia prevention research. The establishment of the global biorepository across the WW‐FINGERS network (network member countries are displayed in green regardless of survey participation) provides a platform for biomarker harmonization, joint analysis and discovery across the globe serving as a unique and transformative resource for the dementia prevention research community. WW‐FINGERS, World‐Wide FINGERS.

Ensuring long‐term sustainability of the global biorepository, including updating methodologies for sample collection, storage, processing, and integration of new sample types, enables equitable, dynamic precision prevention, fostering scientific collaboration, and shaping the future of dementia research worldwide.

## AUTHOR CONTRIBUTIONS

Authors AM, CU, FM, AS and MK have conceptualised and initiated the development of the manuscript. AM, CU, MD, EB, VA conducted data analyses and wrote the first draft. All co‐authors, part of the WW‐FINGERS Network contributed to the manuscript's preparation through providing original data via survey and providing critical review. All authors, including study group co‐authors, reviewed and approved the final version of the manuscript.

## CONFLICT OF INTEREST STATEMENT

M.K. is member of the Combinostics, Biogen, BioArtic, Eli Lilly, and Nestle Advisory Boards; and Speaker for Biogen, Roche, Nestle, and Nutricia. HZ has served at scientific advisory boards and/or as a consultant for Abbvie, Acumen, Alector, Alzinova, ALZpath, Amylyx, Annexon, Apellis, Artery Therapeutics, AZTherapies, Cognito Therapeutics, CogRx, Denali, Eisai, Enigma, LabCorp, Merck Sharp & Dohme, Merry Life, Nervgen, Novo Nordisk, Optoceutics, Passage Bio, Pinteon Therapeutics, Prothena, Quanterix, Red Abbey Labs, reMYND, Roche, Samumed, ScandiBio Therapeutics AB, Siemens Healthineers, Triplet Therapeutics, and Wave, has given lectures sponsored by Alzecure, BioArctic, Biogen, Cellectricon, Fujirebio, LabCorp, Lilly, Novo Nordisk, Oy Medix Biochemica AB, Roche, and WebMD, is a co‐founder of Brain Biomarker Solutions in Gothenburg AB (BBS), which is a part of the GU Ventures Incubator Program, and is a shareholder of CERimmune Therapeutics (outside submitted work). H.B. has been an advisory board member, consultant and/or speaker for Eisai, Eli Lilly, Medicines Australia, NovoNordisk and Roche. Author disclosures are available in the supporting information. H.R.S. and RNM report have had or are receiving research support from Alzheimer's Research Australia as well as Pharmaceutical and Nutraceutical companies including Alector, Alnylam Pharmaceuticals, CWEKPTYLTD, WA, Australia, and Biogen pharmaceuticals. Author disclosures are available in the Supporting Information.

## CONSENT STATEMENT

This study was based on site‐level survey data from multiple studies and randomized controlled trials. Each participating study adhered to its own local ethical approval and consent procedures. As the survey collected only aggregated study‐level information and did not include individual participant data or identifiable information, no additional participant consent was required for the present analysis.

## Supporting information



Supporting Information

Supporting Information

## Collaborators

### 1

World‐Wide FINGERS network

Authors

Dag Aarlsand^1,2^, Daisy Acosta^3^, Panagiotis Alexopoulos^4,5,6^, Ricardo Allegri^7,8^, Kaarin Anstey^9,10,11^, Hidenori Arai^12^, Giuseppe Bellelli^13,14^, Sylvie Belleville^15,16^, G. Bhanuprakash Reddy^17^, Henry Brodaty^18^, Ana Charamelo Baietti^19,20^, Christopher Chen^21,22^, Howard Chertkow^23^, Maria Cusicanqui^24^, Nilton Custodio^25^, Rafael de la Torre^26^, Carolina Delgado^27,28^, Yifeng Du^29,30^, Lissette Duque‐ Peñailillo^31^, Howard Feldman^32^, Giovanni Frisoni^33,34^, Ivonne Z. Jiménez‐ Velázquez^35^, Mei Ling Lim^36,37,38^, Juan J. Llibre‐ Rodriguez^39^, Francisco Lopera^40^, Lucía  Crivelli^41^, Ralph Martins^42,43,44^, Patrizia Mecocci^45,46^, Manuel Montero‐Odasso^47,48,49^, Tiia Ngandu^46,50,51^, Ricardo Nitrini^52^, Haakon Nygaard^53^, Ruth Peters^54,55,56^, Chengxuan Qiu^57,58,59^, Heather Snyder^60^, Gustavo Sevlever^61^, Suzana Shahar^62^, Natalia Soldevila‐ Domenech^63^, Ana Luisa Sosa Ortiz^64^, Elisabeth Stögmann^65^, Ahmet Turan Isik^66^, Barbara Ukropcova^67^, Sebastian  Köhler^68^, Zoltán Vidnyánszky^69^, Eleni Zamba Papanicolaou^70^, Sharon Naismith^71^, Liang‐Kung Chen^72,73,74^, Wei‐Ju Lee^75,76^

Affiliations

^1^Institute of Psychiatry, Psychology and Neuroscience, 16 De Crespigny Park, SE5 8AB King's College London, London, UK

^2^Centre for Age‐Related Medicine, Stavanger University Hospital, Armauer Hansens vei 30, 4011, Stavanger, Norway

^3^Internal Medicine Department, Universidad Nacional Pedro Henriquez Ureña (UNPHU), Geriatric Section, Santo Domingo, Dominican Republic

^4^Patras University Mental Health Services, Department of Medicine, School of Health Sciences, University of Patras, 26504, Rio, Patras, Greece

^5^Global Brain Health Institute, Medical School, Trinity College Dublin, Dublin, Republic of Ireland

^6^Department of Psychiatry and Psychotherapy, Klinikum Rechts der Isar, Faculty of Medicine, Technical University of Munich, Munich, Germany

^7^Department of Cognitive Neurology, Neuropsychology and Neuropsychiatry, Fleni, Buenos Aires, Argentina

^8^Department of Neurosciences, Universidad de la Costa (CUC), Colombia.

^9^UNSW Ageing Futures Institute, University of New South Wales, Australia

^10^School of Psychology, University of New South Wales, Australia

^11^Neuroscience Research Australia, Australia

^12^National Center for Geriatrics and Gerontology, Japan

^13^School of Medicine, University of Milano‐Bicocca, Milan, Italy

^14^Acute Geriatric Unit, IRCCS Foundation San Gerardo, Monza, Italy

^15^Centre de recherche, Institut Universitaire de Gériatrie de Montréal, Montréal, Québec, Canada

^16^Department of psychology, Université de Montréal, Montréal, Québec, Canada

^17^Biochemistry Division, ICMR‐National Institute of Nutrition, Hyderabad, India

^18^Centre for Healthy Brain Ageing, Discipline of Psychiatry and Mental Health, School of Clinical Medicine, University of New South Wales, Sydney, Australia

^19^Clínica de la Memoria. Hospital Británico del Uruguay

^20^Depto de Neuropsicología. Hospital de Clínicas. Universidad de la República, Uruguay

^21^Department of Pharmacology, Yong Loo Lin School of Medicine, National University of Singapore, S117600, Singapore

^22^Memory, Aging and Cognition Center, National University Health Systems, S117600, Singapore

^23^BayCrest Health Sciences, Toronto, Ontario, Canada

^24^Universidad Mayor San Andres, La Paz, Bolivia.

^25^Unidad de diagnóstico de deterioro cognitivo y prevención de demencia, Instituto Peruano de Neurociencias, Lima, Perú.

^26^Department of Medicine and Life Sciences (MELIS), Universitat Pompeu Fabra, Barcelona, Spain.

^27^Departamento de Neurología y Neurocirugía Hospital Clínico Universidad de Chile

^28^Departamento de Neurociencia, Facultad de medicina Universidad de Chile

^29^Department of Neurology, Shandong Provincial Hospital Affiliated to Shandong First Medical University, Jinan, Shandong, P.R. China;

^30^Institute of Brain Science and Brain‐Inspired Research, Shandong First Medical University & Shandong Academy of Medical Sciences, Jinan, Shandong, P.R. China

^31^Cognitive Disorders Unit, Neuromedicenter. Quito—Ecuador

^32^Department of Neurosciences, Alzheimer's Disease Cooperative Study, University of California San Diego, La Jolla, California USA

^33^Division of Geriatrics and Rehabilitation, Memory Centre, University Hospitals of Geneva, Geneva, Switzerland.

^34^Laboratory of Neuroimaging of Aging (LANVIE), University of Geneva, Geneva, Switzerland.

^35^Internal Medicine Department, University of Puerto Rico School of Medicine, Medical Sciences Campus, San Juan, Puerto Rico

^36^The George Institute for Global Health, Sydney, Australia

^37^Falls, Balance and Injury Research Centre, Neuroscience Research Australia, Sydney, Australia

^38^School of Population Health, University of New South Wales, Sydney, Australia

^39^Dementia Research Unit. Medical University of Havana

^40^Grupo de Neurociencias de Antioquia, University of Antioquia, Medellín, Colombia

^41^Department of Cognitive Neurology, Neuropsychology and Neuropsychiatry, Fleni, Buenos Aires, Argentina

^42^UWA School of Medicine, The University of Western Australia, Perth, WA, Australia

^43^Faculty of Medicine and Health Sciences, Macquarie University, Sydney, NSW, Australia

^44^Alzheimer's Research Australia, Perth, WA, Australia

^45^Division of Gerontology and Geriatrics, Department of Medicine and Surgery, University of Perugia, Perugia, Italy

^46^Division of Clinical Geriatrics, Department of Neurobiology, Care Sciences and Society, Karolinska Institutet, Stockholm, Sweden

^47^Gait and Brain Lab, Parkwood Institute, Lawson Research Institute, London, ON, Canada

^48^Division of Geriatric Medicine, Department of Medicine, Schulich School of Medicine & Dentistry, Western University, London, ON, Canada.

^49^Department of Epidemiology and Biostatistics, Schulich School of Medicine & Dentistry, University of Western Ontario, London, ON, Canada.

^50^Department of Public Health, Lifestyles and Living Environments, Finnish Institute for Health and Welfare, Helsinki, Finland

^51^Institute of Public Health and Clinical Nutrition, University of Eastern Finland, Kuopio, Finland

^52^Department of Neurology, University of Sao Paulo, Brazil

^53^Division of Neurology and Djavad Mowafaghian Centre for Brain Helth, University of British Columbia, Vancouver, BC, Canada

^54^The George Institute for Global Health, Australia

^55^School of Population Health, University of New South Wales, Australia

^56^School of Public Health, Imperial College London, UK

^57^Department of Neurology, Shandong Provincial Hospital Affiliated to Shandong First Medical University, Jinan, Shandong, P.R. China;

^58^Institute of Brain Science and Brain‐Inspired Research, Shandong First Medical University & Shandong Academy of Medical Sciences, Jinan, Shandong, P.R. China;

^59^Department of Neurobiology, Care Sciences and Society, Karolinska Institutet, Stockholm, Sweden.

^60^Alzheimer's Association, Chicago, Illinois, USA

^61^Department of Molecular Biology & Neuropathology; Fleni, Buenos Aires, Argentina. Instituto de Neurociencias (CONICET‐Fleni).

^62^Centre for Healthy Aging and Wellness, Faculty of Health Science, Universiti Kebangsaan Malaysia, Kuala Lumpur, Malaysia

^63^Barcelonaβeta Brain Research Center (BBRC), Pasqual Maragall Foundation, Wellington 30, 08005, Barcelona, Spain

^64^Dementias Laboratory, National Institute of Neurology and Neurosurgery, Mexico City, DF 14269, Mexico

^65^Department of Neurology, Medical University Vienna, Vienna, Austria

^66^Unit for Brain Aging and Dementia, Department of Geriatric Medicine, Dokuz Eylul University, School of Medicine, 35340 Balcova, Izmir, Turkey

^67^Center of Physical Activity Research, Biomedical Research Center, Slovak Academy of Sciences, Bratislava, Slovakia

^68^Department of Psychiatry and Neuropsychology, Alzheimer Centrum Limburg, Mental Health and Neuroscience Research Institute (MHeNs), Maastricht University, Maastricht, The Netherlands

^69^Brain Imaging Centre, HUN‐REN Research Centre for Natural Sciences, Budapest, Hungary

^70^Department of Neuroepidemiology, The Cyprus Institute of Neurology and Genetics, Nicosia, Cyprus

^71^Healthy Brain Ageing Program, School of Psychology; Brain and Mind Centre, The University of Sydney, & Charles Perkins Centre, Camperdown, Sydney, Australia.

^72^Center for Geriatrics and Gerontology, Taipei Veterans General Hospital, Taipei, Taiwan

^73^Center for Healthy Longevity and Aging Sciences, National Yang Ming Chiao Tung University, Taipei, Taiwan

^74^Taipei Municipal Gan‐Dau Hospital (Managed by Taipei Veterans General Hospital), Taipei, Taiwan

^75^Department of Family Medicine, Taipei Veterans General Hospital Yuanshan Branch, Yi‐Lan, Taiwan

^76^Center for Healthy Longevity and Aging Sciences, National Yang Ming Chiao Tung University, Taipei, Taiwan.
