# Supplementary material for: Developing the World‐Wide FINGERS biorepository for dementia risk reduction trials
Source: Alzheimers Dement. 2026 Nov 9;22(11):e71949. doi: 10.1002/alz.71949 (PMC13746181; doi:10.1002/alz.71949)
Supplement: Supplementary file 2 [file ALZ-22-e71949-s002.pdf]

Data Dictionary Codebook

20/10/2023 16:26

	#	Variable / Field Name	Field Label<br>*Field Note*	Field Attributes (Field Type, Validation, Choices, Calculations, etc.)
**Instrument: Biomarkers and biorepository studies (biomarkers_and_biorepository_studies)**				Enabled as survey
	1	[record_id]	Record ID	text
	2	[descripsurvey]	Section Header: *General information of the study*<br>The WW-FINGERS Biorepository and Biomarker Workgroup seeks to facilitate joint analyses across member cohorts. Therefore, the following information are required: Have the participants in your study given informed consent for sample sharing with other partners? Have the participants in your study given informed consent for data sharing with other partners? {consent} {agreements} Even if your study does not have agreements in place for sharing, we still want to know about your project. In the future, we hope to be able to share information among the network members to facilitate joint analyses. If you would like to provide more information on the sharing possibilities, please write shortly in the text box below (optional): {sharing_text}	descriptive
	3	[agreements]	Does the study have the necessary agreements for collecting and analyzing biomarkers?	yesno, Required<br>1 Yes<br>0 No
	4	[consent]	Does the study have informed consent forms for collecting and analyzing biomarkers?	yesno, Required<br>1 Yes<br>0 No
	5	[sharing_text]	Text about sharing possibilities	notes
	6	[studyname]	Name of the study	text

	7	[country]	Name of the country<br>*In case the study includes several countries, please answer on behalf of the country you represent.*	dropdown, Required

1	Afghanistan
2	Albania
3	Algeria
4	Andorra
5	Angola
6	Antigua and Barbuda
7	Argentina
8	Armenia
9	Australia
10	Austria
11	Azerbaijan
12	Bahamas
13	Bahrain
14	Bangladesh
15	Barbados
16	Belarus
17	Belgium
18	Belize
19	Benin
20	Bhutan
21	Bolivia
22	Bosnia and Herzegovina
23	Botswana
24	Brazil
25	Brunei
26	Bulgaria
27	Burkina Faso
28	Burundi
29	Côte d'Ivoire
30	Cabo Verde
31	Cambodia
32	Cameroon
33	Canada
34	Central African Republic
35	Chad
36	Chile
37	China
38	Colombia
39	Comoros
40	Congo (Congo-Brazzaville)
41	Costa Rica
42	Croatia
43	Cuba
44	Cyprus
45	Czechia (Czech Republic)
46	Democratic Republic of the Congo
47	Denmark
48	Djibouti
49	Dominica
50	Dominican Republic
51	Ecuador
52	Egypt

53	El Salvador
54	Equatorial Guinea
55	Eritrea
56	Estonia
57	Eswatini (fmr. "Swaziland")
58	Ethiopia
59	Fiji
60	Finland
61	France
62	Gabon
63	Gambia
64	Georgia
65	Germany
66	Ghana
67	Greece
68	Grenada
69	Guatemala
70	Guinea
71	Guinea-Bissau
72	Guyana
73	Haiti
74	Holy See
75	Honduras
76	Hungary
77	Iceland
78	India
79	Indonesia
80	Iran
81	Iraq
82	Ireland
83	Israel
84	Italy
85	Jamaica
86	Japan
87	Jordan
88	Kazakhstan
89	Kenya
90	Kiribati
91	Kuwait
92	Kyrgyzstan
93	Laos
94	Latvia
95	Lebanon
96	Lesotho
97	Liberia
98	Libya
99	Liechtenstein
100	Lithuania
101	Luxembourg
102	Madagascar
103	Malawi
104	Malaysia
105	Maldives

106	Mali
107	Malta
108	Marshall Islands
109	Mauritania
110	Mauritius
111	Mexico
112	Micronesia
113	Moldova
114	Monaco
115	Mongolia
116	Montenegro
117	Morocco
118	Mozambique
119	Myanmar (formerly Burma)
120	Namibia
121	Nauru
122	Nepal
123	Netherlands
124	New Zealand
125	Nicaragua
126	Niger
127	Nigeria
128	North Korea
129	North Macedonia
130	Norway
131	Oman
132	Pakistan
133	Palau
134	Palestine State
135	Panama
136	Papua New Guinea
137	Paraguay
138	Peru
139	Philippines
140	Poland
141	Portugal
142	Qatar
143	Romania
144	Russia
145	Rwanda
146	Saint Kitts and Nevis
147	Saint Lucia
148	Saint Vincent and the Grenadines
149	Samoa
150	San Marino
151	Sao Tome and Principe
152	Saudi Arabia
153	Senegal
154	Serbia
155	Seychelles
156	Sierra Leone
157	Singapore
158	Slovakia

				159 Slovenia<br>160 Solomon Islands<br>161 Somalia<br>162 South Africa<br>163 South Korea<br>164 South Sudan<br>165 Spain<br>166 Sri Lanka<br>167 Sudan<br>168 Suriname<br>169 Sweden<br>170 Switzerland<br>171 Syria<br>196 Taiwan<br>172 Tajikistan<br>173 Tanzania<br>174 Thailand<br>175 Timor-Leste<br>176 Togo<br>177 Tonga<br>178 Trinidad and Tobago<br>179 Tunisia<br>180 Turkey<br>181 Turkmenistan<br>182 Tuvalu<br>183 Uganda<br>184 Ukraine<br>185 United Arab Emirates<br>186 United Kingdom<br>187 United States of America<br>188 Uruguay<br>189 Uzbekistan<br>190 Vanuatu<br>191 Venezuela<br>192 Vietnam<br>193 Yemen<br>194 Zambia<br>195 Zimbabwe
	8	[pi_name]	Name of the Principal Investigator of this study	text, Identifier
	9	[study_stage]	Current study status	dropdown, Required<br>1 Planning<br>2 Ongoing<br>3 Completed
	10	[n_of_study]<br>Show the field ONLY if:<br>[study_stage] = '1' or [study_stage] = '2'	Please select the range that represents the targeted number of participants	radio<br>1 < 100 participants<br>2 100 to 500 participants<br>3 501 to 1000 participants<br>4 >1000 participants<br>Custom alignment: LV
	11	[n_of_study_2]<br>Show the field ONLY if:<br>[study_stage] = '3'	Total number of enrolled participants	text (number, Min: 1), Required

	12	[source_participants]<br><br>Show the field ONLY if:<br>[study_stage] = '1' or [study_stage] = '2' or [study_stage] = '3'	Source of the participants	checkbox<br>1 source_participants___1 Patients from the hospital (i.e Memory clinic)<br>2 source_participants___2 General population<br>3 source_participants___3 Other<br><br>Custom alignment: LV
	13	[specifyother]<br><br>Show the field ONLY if:<br>[source_participants(3)] = '1'	Please specify	text<br>Custom alignment: LH
	14	[cohort_descriotion]<br><br>Show the field ONLY if:<br>[study_stage] = '1' and [study_stage] = '2' and [study_stage] = '3'	Cohort description from your study	checkbox, Required<br>1 cohort_descriotion___1 Healthy subjects<br>2 cohort_descriotion___2 At-risk population<br>3 cohort_descriotion___3 MCI<br>4 cohort_descriotion___4 Alzheimer´s diagnosis<br>5 cohort_descriotion___5 Dementia diagnosis
	15	[collecting]<br><br>Show the field ONLY if:<br>[study_stage] = '2'	Are you currently collecting any biosamples within the framework of your WW-FINGER study?	yesno, Required<br>1 Yes<br>0 No<br><br>Custom alignment: RH
	16	[collecting_3]<br><br>Show the field ONLY if:<br>[study_stage] = '3'	Did you collect any biosamples within the framework of your WW-FINGER study?	yesno, Required<br>1 Yes<br>0 No<br><br>Custom alignment: RH
	17	[collecting_2]<br><br>Show the field ONLY if:<br>[study_stage] = '1'	Are you planning to collect any biosamples within the framework of your WW-FINGER study?	yesno, Required<br>1 Yes<br>0 No<br><br>Custom alignment: RH
	18	[reasons]<br><br>Show the field ONLY if:<br>[collecting] = '0' or [collecting_2] = '0' or [collecting_3] = '0'	Please specify the reason why not.	checkbox<br>1 reasons___1 Because of COVID-19 (restrictions in place)<br>2 reasons___2 Still under ethics committee revision or has been rejected<br>3 reasons___3 Lack of resources<br>4 reasons___4 Other<br>5 reasons___5 We are planning to start collecting but right now, we are not.
	19	[specifyother_2]<br><br>Show the field ONLY if:<br>[reasons(4)] = '1'	Please specify other reasons	text<br>Custom alignment: RH
	20	[support_to_start]<br><br>Show the field ONLY if:<br>[collecting] = '0' or [collecting_2] = '0'	Is there any support you would need from the WW-FINGERS network to start your biorepository?	notes
	21	[apoe]	Do you test for APOE genotype?	yesno<br>1 Yes<br>0 No<br><br>Custom alignment: RH

	22	[fluid_planning]<br><br>Show the field ONLY if:<br>[study_stage] = '1' and [collecting_2] = '1'	Type of sample/fluid you are planning to collect	checkbox<br>7 fluid_planning___7 Whole blood<br>8 fluid_planning___8 RNA (blood)<br>9 fluid_planning___9 Platelets (blood)<br>1 fluid_planning___1 Plasma<br>2 fluid_planning___2 Serum<br>3 fluid_planning___3 CSF<br>4 fluid_planning___4 Urine<br>5 fluid_planning___5 Saliva<br>6 fluid_planning___6 Other
	23	[other_fluid_planning]<br><br>Show the field ONLY if:<br>[fluid_planning(6)] = '1'	Which other type of fluid	text
	24	[storage_temp]<br><br>Show the field ONLY if:<br>[collecting] = '1' or ([collecting] = '0' and ([reasons(5)] = '1' or [reasons(1)] = '1' or [reasons(2)] = '1'))	Section Header: *Biorepository*<br>At which temperature will the samples be stored?<br>*Multiple options allowed*	checkbox<br>1 storage_temp___1 -20°C<br>2 storage_temp___2 -80°C<br>3 storage_temp___3 Other
	25	[storage_temp_other]<br><br>Show the field ONLY if:<br>[storage_temp(3)] = '1'	Please, specify	text (number, Min: -200, Max: +100)
	26	[storage_temp_2]<br><br>Show the field ONLY if:<br>[collecting] = '1' or ([collecting] = '0' and ([reasons(5)] = '1' or [reasons(1)] = '1' or [reasons(2)] = '1'))	Please, provide any additional information on storage temperatures if needed	text (number, Min: -200, Max: +100)
	27	[storage_time]<br><br>Show the field ONLY if:<br>[collecting] = '1' or ([collecting] = '0' and ([reasons(5)] = '1' or [reasons(1)] = '1' or [reasons(2)] = '1'))	For how long will the samples be stored after study completion (years)?	dropdown<br>1 < 5<br>2 5-10<br>3 10-15<br>4 > 15
	28	[storage_1]<br><br>Show the field ONLY if:<br>[collecting] = '1' or ([collecting] = '0' and ([reasons(5)] = '1' or [reasons(1)] = '1' or [reasons(2)] = '1'))	Are the samples collected during the study stored in the study's own facilities?	yesno<br>1 Yes<br>0 No
	29	[storage_1_no]<br><br>Show the field ONLY if:<br>[storage_1] = '0'	If "no", where are the samples stored:	checkbox<br>1 storage_1_no___1 Biobank (outsourced)<br>2 storage_1_no___2 Other
	30	[storage_1_other]<br><br>Show the field ONLY if:<br>[storage_1_no(2)] = '1'	Please specify which other type of facility	text
	31	[ongoingrecruitment]<br><br>Show the field ONLY if:<br>[study_stage] = '2' and [collecting] = '1'	You chose the current study status "ongoing". The following information can be given as approximates: When did you start collecting the biosamples When are you planning to finish the collection of biosamples Types of biosamples you are collecting/planning to collect {startrecruitment_ongoing} {finishrecruitment_ongoing} {fluid_2}	descriptive
	32	[startrecruitment_ongoing]<br><br>Show the field ONLY if:<br>[collecting] = '1' or [study_stage] = '2'	When you started the collection of the biosamples? (This can be an approximate date)	text (date_dmy)
	33	[finishrecruitment_ongoing]<br><br>Show the field ONLY if:<br>[collecting] = '1' or [study_stage] = '2'	When are you planning to complete the collection of the biosamples? (This can be an approximate date)	text (date_dmy)

	34	[fluid_2]	Which other type of biosample	checkbox<br>7 | fluid_2___7 | Whole blood<br>8 | fluid_2___8 | RNA (blood)<br>9 | fluid_2___9 | Platelets (blood)<br>1 | fluid_2___1 | Plasma<br>2 | fluid_2___2 | Serum<br>3 | fluid_2___3 | CSF<br>4 | fluid_2___4 | Urine<br>5 | fluid_2___5 | Saliva<br>6 | fluid_2___6 | Other
	35	[other_ongoing]<br><br>Show the field ONLY if:<br>[fluid_2(6)] = '1'	Please specify which other type of biosample	text
	36	[ongoingrecruitment_no]<br><br>Show the field ONLY if:<br>[study_stage] = '2' and [collecting] = '0' and ([reasons(5)] = '1' or [reasons(1)] = '1' or [reasons(2)] = '1')	You chose the current study status "ongoing". The following information can be given as approximates: When are you planning to start collecting the biosamples When are you planning to finish the collection of biosamples Types of biosamples you are planning to collect {startrecruitment_ongoing_no} {finishrecruitment_ongoing_no} {fluid_2_no}	descriptive
	37	[startrecruitment_ongoing_no]<br><br>Show the field ONLY if:<br>[study_stage] = '2' and [collecting] = '0' and ([reasons(5)] = '1' or [reasons(1)] = '1' or [reasons(2)] = '1')	When you plan to start the collection of the biosamples? (This can be an approximate date)	text (date_dmy)
	38	[finishrecruitment_ongoing_no]<br><br>Show the field ONLY if:<br>[study_stage] = '2' and [collecting] = '0' and ([reasons(5)] = '1' or [reasons(1)] = '1' or [reasons(2)] = '1')	When are you planning to complete the collection of the biosamples? (This can be an approximate date)	text (date_dmy)
	39	[fluid_2_no]<br><br>Show the field ONLY if:<br>[study_stage] = '2' and [collecting] = '0' and ([reasons(5)] = '1' or [reasons(1)] = '1' or [reasons(2)] = '1')	Which other type of biosample	checkbox<br>7 | fluid_2_no___7 | Whole blood<br>8 | fluid_2_no___8 | RNA (blood)<br>9 | fluid_2_no___9 | Platelets (blood)<br>1 | fluid_2_no___1 | Plasma<br>2 | fluid_2_no___2 | Serum<br>3 | fluid_2_no___3 | CSF<br>4 | fluid_2_no___4 | Urine<br>5 | fluid_2_no___5 | Saliva<br>6 | fluid_2_no___6 | Other
	40	[other_ongoing_no]<br><br>Show the field ONLY if:<br>[fluid_2_no(6)] = '1'	Please specify which other type of biosample	text
	41	[blood_samples]<br><br>Show the field ONLY if:<br>[fluid_2(7)] = '1' or [fluid_2(1)] = '1' or [fluid_2(2)] = '1' or [fluid_2(8)] = '1' or [fluid_2(9)] = '1'	BLOOD SAMPLES	descriptive
	42	[ongoing_collect_blood]<br><br>Show the field ONLY if:<br>[fluid_2(7)] = '1' or [fluid_2(1)] = '1' or [fluid_2(2)] = '1' or [fluid_2(8)] = '1' or [fluid_2(9)] = '1'	Is blood collected from the whole cohort?<br>*Multiple options allowed*	checkbox<br>1 | ongoing_collect_blood___1 | Yes<br>2 | ongoing_collect_blood___2 | No

	43	[ongoing_collect_blood_info]<br><br>Show the field ONLY if:<br>[ongoing_collect_blood(2)] = '1'	Please provide some basic information on the subcohort for blood sample collection (estimated): How big is the subcohort (number)? What is the age range of the subcohort? % of female sex {ongoing_sub_blood_size} Min age {ongoing_sub_blood_age1} Max age {ongoing_sub_blood_age2} {ongoing_sub_blood_sex} In case you want to describe the subcohort in more detail, please use the text box below: {ongoing_sub_blood_text}	descriptive
	44	[ongoing_sub_blood_size]	blood subcohort size	text (number, Min: 1, Max: 5000)
	45	[ongoing_sub_blood_age1]	Range of age sub blood Minumum age	text (integer, Min: 10, Max: 110)
	46	[ongoing_sub_blood_age2]	Range of age sub blood Maximum age	text (integer, Min: 10, Max: 110)
	47	[ongoing_sub_blood_sex]	gender (female) sub blood %	text (number, Min: 0, Max: 100)
	48	[ongoing_sub_blood_text]		notes
	49	[plasma_ongoing_details]<br><br>Show the field ONLY if:<br>[fluid_2(1)] = '1' or [fluid_2(2)] = '1' or [fluid_2(7)] = '1' or [fluid_2(8)] = '1' or [fluid_2(9)] = '1'	Please select the characteristics of the blood sample When the samples were collected, were the subjects fasting? {fasting_ongoin_blood} Time of the day when the blood sample was collected {timeofday_blood_ongoing} Sampling every {interval_ongoingblood} months The time between a blood sample and Neuroimaging (months) {imaging_blood} Neuropsychological tests (months) {cogntest_blood} Type of anticoagulant used for the blood samples (only applicable for plasma) {anticoagulant_ongoing}	descriptive
	50	[anticoagulant_ongoing]	Anticoagulant	checkbox<br>1 | anticoagulant_ongoing___1 | EDTA<br>2 | anticoagulant_ongoing___2 | Citrate<br>3 | anticoagulant_ongoing___3 | Heparin<br>4 | anticoagulant_ongoing___4 | Other
	51	[otheranticoag_ongoing]<br><br>Show the field ONLY if:<br>[anticoagulant_ongoing(4)] = '1'	Which other type of anticoagulant	text
	52	[fasting_ongoin_blood]	fasting blood	yesno<br>1 | Yes<br>0 | No
	53	[timeofday_blood_ongoing]	timeofday	radio<br>1 | AM only<br>2 | PM only<br>3 | non-specific
	54	[interval_ongoingblood]	interval	dropdown<br>1 | 0-6<br>2 | 6-12<br>3 | >12
	55	[imaging_blood]	neuroimaging blood	dropdown<br>1 | 0-3<br>2 | 3-6<br>3 | 6-9<br>4 | 9-12
	56	[cogntest_blood]	neuropsychological tests blood	dropdown<br>1 | 0-3<br>2 | 3-6<br>3 | 6-9<br>4 | 9-12
	57	[csf_samples]<br><br>Show the field ONLY if:<br>[fluid_2(3)] = '1'	CSF SAMPLES	descriptive
	58	[ongoing_collect_csf]<br><br>Show the field ONLY if:<br>[fluid_2(3)] = '1'	Is CSF collected from the whole cohort?	yesno<br>1 | Yes<br>0 | No

	59	[ongoing_collect_csf_info]<br><br>Show the field ONLY if:<br>[ongoing_collect_csf] = '0'	Please provide some basic information on the subcohort for CSF sample collection (estimated): How big is the subcohort (number)? What is the age range of the subcohort? % of female sex {ongoing_sub_csf_size} Min age {ongoing_sub_csf_age1} Max age {ongoing_sub_csf_age2} {ongoing_sub_csf_sex} In case you want to describe the subcohort in more detail, please use the text box below: {ongoing_sub_csf_text}	descriptive
	60	[ongoing_sub_csf_size]	csf subcohort size	text (number, Min: 1, Max: 5000)
	61	[ongoing_sub_csf_age1]	Range of age sub csf Minumum age	text (integer, Min: 10, Max: 110)
	62	[ongoing_sub_csf_age2]	Range of age sub csf Maximum age	text (integer, Min: 10, Max: 110)
	63	[ongoing_sub_csf_sex]	gender (female) sub csf %	text (number, Min: 0, Max: 100)
	64	[ongoing_sub_csf_text]		notes
	65	[csf_ongoing_details]<br><br>Show the field ONLY if:<br>[study_stage] = '2' and [fluid_2(3)] = '1'	Please select the characteristics of the CSF sample Time of the day when the CSF sample was collected {timeofday_csfongoing} Sampling every {interval_ongoingcsf} months The time between a CSF sample and Neuroimaging (months) {imaging_csf} Neuropsychological tests (months) {cogntest_csf}	descriptive
	66	[interval_ongoingcsf]	interval	dropdown<br>1 | 0-6<br>2 | 6-12<br>3 | >12
	67	[timeofday_csfongoing]	timeofday	radio<br>1 | AM only<br>2 | PM only<br>3 | non-specific
	68	[imaging_csf]	neuroimaging csf	dropdown<br>1 | 0-3<br>2 | 3-6<br>3 | 6-9<br>4 | 9-12
	69	[cogntest_csf]	neuropsychological tests csf	dropdown<br>1 | 0-3<br>2 | 3-6<br>3 | 6-9<br>4 | 9-12
	70	[urine_sample]<br><br>Show the field ONLY if:<br>[fluid_2(4)] = '1'	URINE SAMPLES	descriptive
	71	[ongoing_collect_urine]<br><br>Show the field ONLY if:<br>[fluid_2(4)] = '1'	Is urine collected from the whole cohort?	yesno<br>1 | Yes<br>0 | No
	72	[ongoing_collect_urine_info]<br><br>Show the field ONLY if:<br>[ongoing_collect_urine] = '0'	Please provide some basic information on the subcohort for urine sample collection (estimated): How big is the subcohort (number)? What is the age range of the subcohort? % of female sex {ongoing_sub_urine_size} Min age {ongoing_sub_urine_age1} Max age {ongoing_sub_urine_age2} {ongoing_sub_urine_sex} In case you want to describe the subcohort in more detail, please use the text box below: {ongoing_sub_urine_text}	descriptive
	73	[ongoing_sub_urine_size]	urine subcohort size	text (number, Min: 1, Max: 5000)
	74	[ongoing_sub_urine_age1]	Range of age sub urine Minumum age	text (integer, Min: 10, Max: 110)
	75	[ongoing_sub_urine_age2]	Range of age sub urine Maximum age	text (integer, Min: 10, Max: 110)
	76	[ongoing_sub_urine_sex]	gender (female) sub urine %	text (number, Min: 0, Max: 100)
	77	[ongoing_sub_urine_text]		notes

	78	[urine_ongoing_details]<br><br>Show the field ONLY if:<br>[study_stage] = '2' and [fluid_2(4)] = '1'	Please select the characteristics of the urine sample Time of the day when the urine sample was collected {timeofday_urine_ongoing} Please specify the sampling interval/frequency {interval_ongoingurine} The time between a urine sample and Neuroimaging (months) {imaging_urine} Neuropsychological tests (months) {cogntest_urine}	descriptive
	79	[timeofday_urine_ongoing]	timeofday	radio<br>1 AM only<br>2 PM only<br>3 non-specific
	80	[interval_ongoingurine]	interval	text
	81	[imaging_urine]	neuroimaging urine	dropdown<br>1 0-3<br>2 3-6<br>3 6-9<br>4 9-12
	82	[cogntest_urine]	neuropsychological tests urine	dropdown<br>1 0-3<br>2 3-6<br>3 6-9<br>4 9-12
	83	[saliva_samples]<br><br>Show the field ONLY if:<br>[fluid_2(5)] = '1'	SALIVA SAMPLES	descriptive
	84	[ongoing_collect_saliva]<br><br>Show the field ONLY if:<br>[fluid_2(5)] = '1'	Is saliva collected from the whole cohort?	yesno<br>1 Yes<br>0 No
	85	[ongoing_collect_saliva_info]<br><br>Show the field ONLY if:<br>[ongoing_collect_saliva] = '0'	Please provide some basic information on the subcohort for saliva sample collection (estimated): How big is the subcohort (number)? What is the age range of the subcohort? % of female sex {ongoing_sub_saliva_size} Min age {ongoing_sub_saliva_age1} Max age {ongoing_sub_saliva_age2} {ongoing_sub_saliva_sex} In case you want to describe the subcohort in more detail, please use the text box below: {ongoing_sub_saliva_text}	descriptive
	86	[ongoing_sub_saliva_size]	saliva subcohort size	text (number, Min: 1, Max: 5000)
	87	[ongoing_sub_saliva_age1]	Range of age sub saliva Minumum age	text (integer, Min: 10, Max: 110)
	88	[ongoing_sub_saliva_age2]	Range of age sub saliva Maximum age	text (integer, Min: 10, Max: 110)
	89	[ongoing_sub_saliva_sex]	gender (female) sub saliva %	text (number, Min: 0, Max: 100)
	90	[ongoing_sub_saliva_text]		notes
	91	[saliva_ongoing_details]<br><br>Show the field ONLY if:<br>[study_stage] = '2' and [fluid_2(5)] = '1'	Please select the characteristics of the saliva sample Time of the day when the saliva sample was collected {timeofday_saliva_ongoing} Please, specify the sampling interval/frequency {interval_ongoingsaliva} The time between a saliva sample and Neuroimaging (months) {imaging_saliva} Neuropsychological tests (months) {cogntest_saliva}	descriptive
	92	[timeofday_saliva_ongoing]	timeofday	radio<br>1 AM only<br>2 PM only<br>3 non-specific
	93	[interval_ongoingsaliva]	interval	text
	94	[imaging_saliva]	neuroimaging saliva	dropdown<br>1 0-3<br>2 3-6<br>3 6-9<br>4 9-12

	95	[cogntest_saliva]	neuropsychological tests saliva	dropdown<br>1 0-3<br>2 3-6<br>3 6-9<br>4 9-12
	96	[storage_temp_compl]<br>Show the field ONLY if:<br>[collecting_3] = '1'	At which temperature are the samples stored?<br>*Multiple options allowed*	checkbox<br>1 storage_temp_compl___1 -20°C<br>2 storage_temp_compl___2 -80°C<br>3 storage_temp_compl___3 Other
	97	[storage_temp_other_compl]<br>Show the field ONLY if:<br>[storage_temp_compl(3)] = '1'	Please, specify	text (number, Min: -200, Max: +100)
	98	[storage_temp_compl_2]<br>Show the field ONLY if:<br>[collecting_3] = '1'	Please, provide any additional information on storage temperatures if needed	text (number, Min: -200, Max: +100)
	99	[storage_time_compl]<br>Show the field ONLY if:<br>[collecting_3] = '1'	For how long will the samples be stored after study completion (years)?	dropdown<br>1 < 5<br>2 5-10<br>3 10-15<br>4 > 15
	100	[storage_compl]<br>Show the field ONLY if:<br>[collecting_3] = '1'	Are the samples collected during the study stored in the study's own facilities?	yesno<br>1 Yes<br>0 No
	101	[storage_no_compl]<br>Show the field ONLY if:<br>[storage_compl] = '0'	If "no", where are the samples stored:	checkbox<br>1 storage_no_compl___1 Biobank (outsourced)<br>2 storage_no_compl___2 Other
	102	[storage_other_compl]<br>Show the field ONLY if:<br>[storage_no_compl(2)] = '1'	Please specify which other type of facility	text
	103	[pop]<br>Show the field ONLY if:<br>[study_stage] = '3' and [collecting_3] = '1'	We would like to know more about the study population. Range of age % of the female sex Ethnicity Minimum age {agerange} Maximum age {agerange_max} {female} Please choose the ethnic group(s) that are part of your study population. {ethnicity}	descriptive
	104	[agerange]<br>Show the field ONLY if:<br>[study_stage] = '3'	Range of age Minumum age	text (integer, Min: 10, Max: 110)
	105	[agerange_max]<br>Show the field ONLY if:<br>[study_stage] = '3'	Range of age Maximum age	text (integer, Min: 10, Max: 110)
	106	[female]<br>Show the field ONLY if:<br>[study_stage] = '3'	gender (female) %	text (number)
	107	[ethnicity]<br>Show the field ONLY if:<br>[study_stage] = '3'	Please choose the ethnic group(s) that are part of your study population.	checkbox<br>1 ethnicity___1 White<br>2 ethnicity___2 Asian<br>6 ethnicity___6 Middle Eastern<br>3 ethnicity___3 Black African<br>7 ethnicity___7 Caribbean<br>8 ethnicity___8 Hispanic or Latino<br>9 ethnicity___9 Pacific Islander<br>4 ethnicity___4 Mixed<br>5 ethnicity___5 Other
	108	[other_ethspecify]<br>Show the field ONLY if:<br>[ethnicity(5)] = '1' and [study_stage] = '3'	If other ethnicities, please specify	text

	109	[finishcollection]<br><br>Show the field ONLY if:<br>[study_stage] = '3' and [collecting_3] = '1'	You chose the current study status "completed". Please, provide the following information Biosample collection Participants who provided samples for the biobank Type of fluid/ Biosample When did the collection start? {finish_startdate} When did the collection finish? {finish_date} Total number {totalncollected} Which type of samples you collected? {fluid}	descriptive
	110	[finish_startdate]	Start date	text (date_dmy)
	111	[finish_date]	finish date	text (date_dmy)
	112	[totalncollected]	number	text (number, Min: 0)
	113	[fluid]	Type of sample	checkbox<br>7 | fluid___7 | Whole blood<br>8 | fluid___8 | RNA (blood)<br>9 | fluid___9 | Platelets (blood)<br>1 | fluid___1 | Plasma<br>2 | fluid___2 | Serum<br>3 | fluid___3 | CSF<br>4 | fluid___4 | Urine<br>5 | fluid___5 | Saliva<br>6 | fluid___6 | Other
	114	[other_fluid]<br><br>Show the field ONLY if:<br>[fluid(6)] = '1'	Which other type of fluid	text
	115	[blood_samples_2]<br><br>Show the field ONLY if:<br>[fluid(7)] = '1' or [fluid(1)] = '1' or [fluid(2)] = '1' or [fluid(8)] = '1' or [fluid(9)] = '1'	BLOOD SAMPLES	descriptive
	116	[compl_collect_blood]<br><br>Show the field ONLY if:<br>[fluid(7)] = '1' or [fluid(1)] = '1' or [fluid(2)] = '1' or [fluid(8)] = '1' or [fluid(9)] = '1'	Was blood collected from the whole cohort?<br>*Multiple options allowed*	checkbox<br>1 | compl_collect_blood___1 | Yes<br>2 | compl_collect_blood___2 | No
	117	[compl_collect_blood_info]<br><br>Show the field ONLY if:<br>[compl_collect_blood(2)] = '1'	Please provide some basic information on the subcohort for blood sample collection (estimated): How big is the subcohort (number)? What is the age range of the subcohort? % of female sex {compl_sub_blood_size} Min age {compl_sub_blood_age1} Max age {compl_sub_blood_age2} {compl_sub_blood_sex} In case you want to describe the subcohort in more detail, please use the text box below: {compl_sub_blood_text}	descriptive
	118	[compl_sub_blood_size]	blood subcohort size	text (number, Min: 1, Max: 5000)
	119	[compl_sub_blood_age1]	Range of age sub blood Minumum age	text (integer, Min: 10, Max: 110)
	120	[compl_sub_blood_age2]	Range of age sub blood Maximum age	text (integer, Min: 10, Max: 110)
	121	[compl_sub_blood_sex]	gender (female) sub blood %	text (number, Min: 0, Max: 100)
	122	[compl_sub_blood_text]		notes
	123	[blood_complet_details]<br><br>Show the field ONLY if:<br>[study_stage] = '3' and ([fluid(1)] = '1' or [fluid(7)] = '1' or [fluid(2)] = '1' or [fluid(8)] = '1' or [fluid(9)] = '1')	Please select the characteristics of the blood sample When the samples were collected, were the subjects fasting? {blood_fasting_compl} Time of the day when the blood sample was collected {timeofday} Sampling every {interval_compl_blood} months The time between a blood sample and Neuroimaging (months) {imaging_blood_compl} Neuropsychological tests (months) {cogntest_blood_compl} Type of anticoagulant used for the blood samples (only applicable for plasma) {anticoagulant}	descriptive
	124	[blood_fasting_compl]	fasting blood	yesno<br>1 | Yes<br>0 | No

	125	[anticoagulant]	Anticoagulant	checkbox<br>1 anticoagulant___1 EDTA<br>2 anticoagulant___2 Citrate<br>3 anticoagulant___3 Heparin<br>4 anticoagulant___4 Other
	126	[other_anticoag]<br>Show the field ONLY if:<br>[anticoagulant(4)] = '1'	Which other type of anticoagulant	text
	127	[timeofday]	timeofday	radio<br>1 AM only<br>2 PM only<br>3 non-specific
	128	[interval_compl_blood]	interval	dropdown<br>1 0-6<br>2 6-12<br>3 >12
	129	[imaging_blood_compl]	neuroimaging blood	dropdown<br>1 0-6<br>2 6-12<br>3 >12
	130	[cogntest_blood_compl]	neuropsychological tests blood	dropdown<br>1 0-6<br>2 6-12<br>3 >12
	131	[csf_samples_2]<br>Show the field ONLY if:<br>[fluid(3)] = '1'	CSF SAMPLES	descriptive
	132	[compl_collect_csf]<br>Show the field ONLY if:<br>[fluid(3)] = '1'	Was CSF collected from the whole cohort?	yesno<br>1 Yes<br>0 No
	133	[compl_collect_csf_info]<br>Show the field ONLY if:<br>[compl_collect_csf] = '0'	Please provide some basic information on the subcohort for CSF sample collection (estimated): How big is the subcohort (number)? What is the age range of the subcohort? % of female sex {compl_sub_csf_size} Min age {compl_sub_csf_age1} Max age {compl_sub_csf_age2} {compl_sub_csf_sex} In case you want to describe the subcohort in more detail, please use the text box below: {compl_sub_csf_text}	descriptive
	134	[compl_sub_csf_size]	csf subcohort size	text (number, Min: 1, Max: 5000)
	135	[compl_sub_csf_age1]	Range of age sub csf Minumum age	text (integer, Min: 10, Max: 110)
	136	[compl_sub_csf_age2]	Range of age sub csf Maximum age	text (integer, Min: 10, Max: 110)
	137	[compl_sub_csf_sex]	gender (female) sub csf %	text (number, Min: 0, Max: 100)
	138	[compl_sub_csf_text]		notes
	139	[csfcomplete_details]<br>Show the field ONLY if:<br>[study_stage] = '3' and [fluid (3)] = '1'	Please select the characteristics of the CSF sample Time of the day when the CSF sample was collected {csf_timeofday} Sampling every {interval_compl_csf} months The time between a CSF sample and Neuroimaging (months) {imaging_csf_compl} Neuropsychological tests (months) {cogntest_csf_compl}	descriptive
	140	[csf_timeofday]	Time of the day for CSF	radio<br>1 AM only<br>2 PM only<br>3 non-specific
	141	[interval_compl_csf]	interval	dropdown<br>1 0-6<br>2 6-12<br>3 >12

	142	[imaging_csf_compl]	neuroimaging csf	dropdown<br>1 0-6<br>2 6-12<br>3 >12
	143	[cogntest_csf_compl]	neuropsychological tests csf	dropdown<br>1 0-6<br>2 6-12<br>3 >12
	144	[urine_samples_2]<br><br>Show the field ONLY if:<br>[fluid(4)] = '1'	URINE SAMPLES	descriptive
	145	[compl_collect_urine]<br><br>Show the field ONLY if:<br>[fluid(4)] = '1'	Was urine collected from the whole cohort?	yesno<br>1 Yes<br>0 No
	146	[compl_collect_urine_info]<br><br>Show the field ONLY if:<br>[compl_collect_urine] = '0'	Please provide some basic information on the subcohort for urine sample collection (estimated): How big is the subcohort (number)? What is the age range of the subcohort? % of female sex {compl_sub_urine_size} Min age {compl_sub_urine_age1} Max age {compl_sub_urine_age2} {compl_sub_urine_sex} In case you want to describe the subcohort in more detail, please use the text box below: {compl_sub_urine_text}	descriptive
	147	[compl_sub_urine_size]	urine subcohort size	text (number, Min: 1, Max: 5000)
	148	[compl_sub_urine_age1]	Range of age sub urine Minumum age	text (integer, Min: 10, Max: 110)
	149	[compl_sub_urine_age2]	Range of age sub urine Maximum age	text (integer, Min: 10, Max: 110)
	150	[compl_sub_urine_sex]	gender (female) sub urine %	text (number, Min: 0, Max: 100)
	151	[compl_sub_urine_text]		notes
	152	[urine_complete_details]<br><br>Show the field ONLY if:<br>[study_stage] = '3' and [fluid (4)] = '1'	Please select the characteristics of the urine sample Time of the day when the urine sample was collected {urine_timeofday} Please, specify the sampling interval/frequency {interval_compl_urine} The time between a urine sample and Neuroimaging (months) {imaging_urine_compl} Neuropsychological tests (months) {cogntest_urine_compl}	descriptive
	153	[urine_timeofday]	Time of the day for urine	radio<br>1 AM only<br>2 PM only<br>3 non-specific
	154	[interval_compl_urine]	interval	text
	155	[imaging_urine_compl]	neuroimaging urine	dropdown<br>1 0-6<br>2 6-12<br>3 >12
	156	[cogntest_urine_compl]	neuropsychological tests urine	dropdown<br>1 0-6<br>2 6-12<br>3 >12
	157	[saliva_samples_2]<br><br>Show the field ONLY if:<br>[fluid(5)] = '1'	SALIVA SAMPLES	descriptive
	158	[compl_collect_saliva]<br><br>Show the field ONLY if:<br>[fluid(5)] = '1'	Was saliva collected from the whole cohort?	yesno<br>1 Yes<br>0 No

	159	[compl_collect_saliva_info]<br><br>Show the field ONLY if:<br>[compl_collect_saliva] = '0'	Please provide some basic information on the subcohort for saliva sample collection (estimated): How big is the subcohort (number)? What is the age range of the subcohort? % of female sex {compl_sub_saliva_size} Min age {compl_sub_saliva_age1} Max age {compl_sub_saliva_age2} {compl_sub_saliva_sex} In case you want to describe the subcohort in more detail, please use the text box below: {compl_sub_saliva_text}	descriptive
	160	[compl_sub_saliva_size]	saliva subcohort size	text (number, Min: 1, Max: 5000)
	161	[compl_sub_saliva_age1]	Range of age sub saliva Minumum age	text (integer, Min: 10, Max: 110)
	162	[compl_sub_saliva_age2]	Range of age sub saliva Maximum age	text (integer, Min: 10, Max: 110)
	163	[compl_sub_saliva_sex]	gender (female) sub saliva %	text (number, Min: 0, Max: 100)
	164	[compl_sub_saliva_text]		notes
	165	[saliva_completedetails]<br><br>Show the field ONLY if:<br>[study_stage] = '3' and [fluid (5)] = '1'	Please select the characteristics of the saliva sample Time of the day when the saliva sample was collected {saliva_timeofday} Please, specify the sampling interval/frequency {interval_compl_saliva} The time between a saliva sample and Neuroimaging (months) {imaging_saliva_compl} Neuropsychological tests (months) {cogntest_saliva_compl}	descriptive
	166	[saliva_timeofday]	Time of the day for saliva	radio<br>1 AM only<br>2 PM only<br>3 non-specific
	167	[interval_compl_saliva]	interval	text
	168	[imaging_saliva_compl]	neuroimaging saliva	dropdown<br>1 0-6<br>2 6-12<br>3 >12
	169	[cogntest_saliva_compl]	neuropsychological tests saliva	dropdown<br>1 0-6<br>2 6-12<br>3 >12
	170	[wb_bm_planning]<br><br>Show the field ONLY if:<br>[fluid_planning(7)] = '1'	Section Header: *Are you planning* to measure any of these biomarkers?*<br><br>Whole blood	checkbox<br>1 wb_bm_planning___1 Aβ<br>2 wb_bm_planning___2 Total Tau<br>3 wb_bm_planning___3 p-Tau<br>4 wb_bm_planning___4 NfL<br>5 wb_bm_planning___5 GFAP<br>6 wb_bm_planning___6 Prote-omics<br>7 wb_bm_planning___7 Metab-olomics<br>8 wb_bm_planning___8 Epigen-omics<br>9 wb_bm_planning___9 Lipid-omics<br>10 wb_bm_planning___10 GWAS<br>11 wb_bm_planning___11 APOE
	171	[plasma_bm_planning]<br><br>Show the field ONLY if:<br>[fluid_planning(1)] = '1'	Plasma	checkbox<br>1 plasma_bm_planning___1 Aβ<br>2 plasma_bm_planning___2 Total Tau<br>3 plasma_bm_planning___3 p-Tau<br>4 plasma_bm_planning___4 NfL<br>5 plasma_bm_planning___5 GFAP<br>6 plasma_bm_planning___6 Prote-omics<br>7 plasma_bm_planning___7 Metab-olomics<br>8 plasma_bm_planning___8 Epigen-omics<br>9 plasma_bm_planning___9 Lipid-omics<br>10 plasma_bm_planning___10 GWAS<br>11 plasma_bm_planning___11 APOE

	172	[serum_bm_planning]<br><br>Show the field ONLY if:<br>[fluid_planning(2)] = '1'	Serum	checkbox<br>1 serum_bm_planning___1 Aβ<br>2 serum_bm_planning___2 Total Tau<br>3 serum_bm_planning___3 p-Tau<br>4 serum_bm_planning___4 NfL<br>5 serum_bm_planning___5 GFAP<br>6 serum_bm_planning___6 Prote-omics<br>7 serum_bm_planning___7 Metab-olomics<br>8 serum_bm_planning___8 Epigen-omics<br>9 serum_bm_planning___9 Lipid-omics<br>10 serum_bm_planning___10 GWAS<br>11 serum_bm_planning___11 APOE
	173	[csf_bm_planning]<br><br>Show the field ONLY if:<br>[fluid_planning(3)] = '1'	CSF	checkbox<br>1 csf_bm_planning___1 Aβ<br>2 csf_bm_planning___2 Total Tau<br>3 csf_bm_planning___3 p-Tau<br>4 csf_bm_planning___4 NfL<br>5 csf_bm_planning___5 GFAP<br>6 csf_bm_planning___6 Prote-omics<br>7 csf_bm_planning___7 Metab-olomics<br>8 csf_bm_planning___8 Epigen-omics<br>9 csf_bm_planning___9 Lipid-omics<br>10 csf_bm_planning___10 GWAS<br>11 csf_bm_planning___11 APOE
	174	[urine_bm_planning]<br><br>Show the field ONLY if:<br>[fluid_planning(4)] = '1'	Urine	checkbox<br>1 urine_bm_planning___1 Aβ<br>2 urine_bm_planning___2 Total Tau<br>3 urine_bm_planning___3 p-Tau<br>4 urine_bm_planning___4 NfL<br>5 urine_bm_planning___5 GFAP<br>6 urine_bm_planning___6 Prote-omics<br>7 urine_bm_planning___7 Metab-olomics<br>8 urine_bm_planning___8 Epigen-omics<br>9 urine_bm_planning___9 Lipid-omics<br>10 urine_bm_planning___10 GWAS<br>11 urine_bm_planning___11 APOE
	175	[saliva_bm_planning]<br><br>Show the field ONLY if:<br>[fluid_planning(5)] = '1'	Saliva	checkbox<br>1 saliva_bm_planning___1 Aβ<br>2 saliva_bm_planning___2 Total Tau<br>3 saliva_bm_planning___3 p-Tau<br>4 saliva_bm_planning___4 NfL<br>5 saliva_bm_planning___5 GFAP<br>6 saliva_bm_planning___6 Prote-omics<br>7 saliva_bm_planning___7 Metab-olomics<br>8 saliva_bm_planning___8 Epigen-omics<br>9 saliva_bm_planning___9 Lipid-omics<br>10 saliva_bm_planning___10 GWAS<br>11 saliva_bm_planning___11 APOE

	176	[other_bm_planning]<br><br>Show the field ONLY if:<br>[fluid_planning(6)] = '1'	Other	checkbox<br>1 other_bm_planning___1 Aβ<br>2 other_bm_planning___2 Total Tau<br>3 other_bm_planning___3 p-Tau<br>4 other_bm_planning___4 NfL<br>5 other_bm_planning___5 GFAP<br>6 other_bm_planning___6 Prote-omics<br>7 other_bm_planning___7 Metab-olomics<br>8 other_bm_planning___8 Epigen-omics<br>9 other_bm_planning___9 Lipid-omics<br>10 other_bm_planning___10 GWAS<br>11 other_bm_planning___11 APOE
	177	[otherbiom_planning]<br><br>Show the field ONLY if:<br>[fluid_planning(1)] = '1' or [fluid_planning(2)] = '1' or [fluid_planning(3)] = '1' or [fluid_planning(4)] = '1' or [fluid_planning(5)] = '1' or [fluid_planning(6)] = '1' or [fluid_planning(7)] = '1' or [fluid_planning(8)] = '1' or [fluid_planning(9)] = '1'	Are you planning to measure any other biomarkers?	text
	178	[including_biomarkers_grant]<br><br>Show the field ONLY if:<br>[fluid_planning(1)] = '1' or [fluid_planning(2)] = '1' or [fluid_planning(3)] = '1' or [fluid_planning(4)] = '1' or [fluid_planning(5)] = '1' or [fluid_planning(6)] = '1' or [fluid_planning(7)] = '1' or [fluid_2_no(1)] = '1' or [fluid_2_no(2)] = '1' or [fluid_2_no(3)] = '1' or [fluid_2_no(4)] = '1' or [fluid_2_no(5)] = '1' or [fluid_2_no(6)] = '1' or [fluid_2_no(7)] = '1' or [fluid_2_no(8)] = '1' or [fluid_2_no(9)] = '1'	*Including biomarkers for which grant applications are planned, i.e., are on a wish-list. Aβ = β Amyloid, p-Tau = Phosphorylated Tau, NfL = Neurofilament light, GFAP = Glial fibrillary acidic protein, GWAS = Genome wide association study, APOE = APOE genotype	descriptive

	179	[ongoing_biomarkers_in_fluids]<br><br>Show the field ONLY if:<br>[study_stage] = '2' and [collecting] = '1'	Section Header: *Biomarkers*<br>Please select the biomarkers measured in different biosamples in the ongoing project and the platform(s) you plan to use to analyze the different markers. Answer options: Yes (ongoing or planned), Wish-list (grant applications planned/pending), No β-Amyloid Total Tau Phosphorylated Tau Glial fibrillary acid protein GWAS Plasma {og_bm_plasma} {og_bm_plasma_2} {og_bm_plasma_3} {og_bm_plasma_4} Serum {og_bm_serum} {og_bm_serum_2} {og_bm_serum_3} {og_bm_serum_4} CSF {og_bm_csf} {og_bm_csf_2} {og_bm_csf_3} {og_bm_csf_4} Urine {og_bm_urine} {og_bm_urine_2} {og_bm_urine_3} {og_bm_urine_4} Saliva {og_bm_saliva} {og_bm_saliva_2} {og_bm_saliva_3} {og_bm_saliva_4} Whole blood {og_bm_wb} Platform(s)* {ongoign_ab} {ongoign_ttau} {ongoign_ptau} {ongoign_gfap} {ongoign_gwas} *Please specify the sample type for each platform Neurofilament light Proteomics Metabolomics Epigenomics Lipidomics Other Plasma {og_bm_plasma_10} {og_bm_plasma_5} {og_bm_plasma_6} {og_bm_plasma_7} {og_bm_plasma_8} {og_bm_plasma_9} {og_bm_plasma_o} Serum {og_bm_serum_10} {og_bm_serum_5} {og_bm_serum_6} {og_bm_serum_7} {og_bm_serum_8} {og_bm_serum_9} {og_bm_serum_o} CSF {og_bm_csf_10} {og_bm_csf_5} {og_bm_csf_6} {og_bm_csf_7} {og_bm_csf_8} {og_bm_csf_9} {og_bm_csf_o} Urine {og_bm_urine_10} {og_bm_urine_5} {og_bm_urine_6} {og_bm_urine_7} {og_bm_urine_8} {og_bm_urine_9} {og_bm_urine_o} Saliva {og_bm_saliva_10} {og_bm_saliva_5} {og_bm_saliva_6} {og_bm_saliva_7} {og_bm_saliva_8} {og_bm_saliva_9} {og_bm_saliva_o} Platform(s)* {ongoign_nfl} {ongoign_prot} {ongoign_lipi} {ongoign_metab} {ongoign_epi} {ongoign_other} *Please specify the sample type for each platform	descriptive
	180	[other_ongoing_2]<br><br>Show the field ONLY if:<br>[fluid_2(6)] = '1'	Please specify the biomarkers measured in other types of biosamples	text
	181	[og_bm_wb]<br><br>Show the field ONLY if:<br>[study_stage] = '2' and [fluid_2(7)] = '1'		radio<br>1 Yes<br>2 Wish-list<br>3 No
	182	[og_bm_plasma]<br><br>Show the field ONLY if:<br>[study_stage] = '2' and [fluid_2(1)] = '1'		radio<br>1 Yes<br>2 Wish-list<br>3 No
	183	[og_bm_plasma_2]<br><br>Show the field ONLY if:<br>[study_stage] = '2' and [fluid_2(1)] = '1'		radio<br>1 Yes<br>2 Wish-list<br>3 No
	184	[og_bm_plasma_3]<br><br>Show the field ONLY if:<br>[study_stage] = '2' and [fluid_2(1)] = '1'		radio<br>1 Yes<br>2 Wish-list<br>3 No
	185	[og_bm_plasma_4]<br><br>Show the field ONLY if:<br>[study_stage] = '2' and [fluid_2(1)] = '1'		radio<br>1 Yes<br>2 Wish-list<br>3 No
	186	[og_bm_plasma_5]<br><br>Show the field ONLY if:<br>[study_stage] = '2' and [fluid_2(1)] = '1'		radio<br>1 Yes<br>2 Wish-list<br>3 No

	187	[og_bm_plasma_6]<br><br>Show the field ONLY if:<br>[study_stage] = '2' and [fluid_2(1)] = '1'		radio<br>1 Yes<br>2 Wish-list<br>3 No
	188	[og_bm_plasma_7]<br><br>Show the field ONLY if:<br>[study_stage] = '2' and [fluid_2(1)] = '1'		radio<br>1 Yes<br>2 Wish-list<br>3 No
	189	[og_bm_plasma_8]<br><br>Show the field ONLY if:<br>[study_stage] = '2' and [fluid_2(1)] = '1'		radio<br>1 Yes<br>2 Wish-list<br>3 No
	190	[og_bm_plasma_9]<br><br>Show the field ONLY if:<br>[study_stage] = '2' and [fluid_2(1)] = '1'		radio<br>1 Yes<br>2 No
	191	[og_bm_plasma_10]<br><br>Show the field ONLY if:<br>[study_stage] = '2' and [fluid_2(1)] = '1'		radio<br>1 Yes<br>2 No
	192	[og_bm_plasma_o]<br><br>Show the field ONLY if:<br>[study_stage] = '2' and [fluid_2(1)] = '1' and [og_bm_plasma_9] = '1'		text
	193	[og_bm_serum]<br><br>Show the field ONLY if:<br>[study_stage] = '2' and [fluid_2(2)] = '1'		radio<br>1 Yes<br>2 Wish-list<br>3 No
	194	[og_bm_serum_2]<br><br>Show the field ONLY if:<br>[study_stage] = '2' and [fluid_2(2)] = '1'		radio<br>1 Yes<br>2 Wish-list<br>3 No
	195	[og_bm_serum_3]<br><br>Show the field ONLY if:<br>[study_stage] = '2' and [fluid_2(2)] = '1'		radio<br>1 Yes<br>2 Wish-list<br>3 No
	196	[og_bm_serum_4]<br><br>Show the field ONLY if:<br>[study_stage] = '2' and [fluid_2(2)] = '1'		radio<br>1 Yes<br>2 Wish-list<br>3 No
	197	[og_bm_serum_5]<br><br>Show the field ONLY if:<br>[study_stage] = '2' and [fluid_2(2)] = '1'		radio<br>1 Yes<br>2 Wish-list<br>3 No
	198	[og_bm_serum_6]<br><br>Show the field ONLY if:<br>[study_stage] = '2' and [fluid_2(2)] = '1'		radio<br>1 Yes<br>2 Wish-list<br>3 No
	199	[og_bm_serum_7]<br><br>Show the field ONLY if:<br>[study_stage] = '2' and [fluid_2(2)] = '1'		radio<br>1 Yes<br>2 Wish-list<br>3 No

	200	[og_bm_serum_8]<br><br>Show the field ONLY if:<br>[study_stage] = '2' and [fluid_2(2)] = '1'		radio<br>1 Yes<br>2 Wish-list<br>3 No
	201	[og_bm_serum_9]<br><br>Show the field ONLY if:<br>[study_stage] = '2' and [fluid_2(2)] = '1'		radio<br>1 Yes<br>2 No
	202	[og_bm_serum_10]<br><br>Show the field ONLY if:<br>[study_stage] = '2' and [fluid_2(2)] = '1'		radio<br>1 Yes<br>2 No
	203	[og_bm_serum_o]<br><br>Show the field ONLY if:<br>[study_stage] = '2' and [fluid_2(2)] = '1' and [og_bm_serum_9] = '1'		text
	204	[og_bm_csf]<br><br>Show the field ONLY if:<br>[study_stage] = '2' and [fluid_2(3)] = '1'		radio<br>1 Yes<br>2 Wish-list<br>3 No
	205	[og_bm_csf_2]<br><br>Show the field ONLY if:<br>[study_stage] = '2' and [fluid_2(3)] = '1'		radio<br>1 Yes<br>2 Wish-list<br>3 No
	206	[og_bm_csf_3]<br><br>Show the field ONLY if:<br>[study_stage] = '2' and [fluid_2(3)] = '1'		radio<br>1 Yes<br>2 Wish-list<br>3 No
	207	[og_bm_csf_4]<br><br>Show the field ONLY if:<br>[study_stage] = '2' and [fluid_2(3)] = '1'		radio<br>1 Yes<br>2 Wish-list<br>3 No
	208	[og_bm_csf_5]<br><br>Show the field ONLY if:<br>[study_stage] = '2' and [fluid_2(3)] = '1'		radio<br>1 Yes<br>2 Wish-list<br>3 No
	209	[og_bm_csf_6]<br><br>Show the field ONLY if:<br>[study_stage] = '2' and [fluid_2(3)] = '1'		radio<br>1 Yes<br>2 Wish-list<br>3 No
	210	[og_bm_csf_7]<br><br>Show the field ONLY if:<br>[study_stage] = '2' and [fluid_2(3)] = '1'		radio<br>1 Yes<br>2 Wish-list<br>3 No
	211	[og_bm_csf_8]<br><br>Show the field ONLY if:<br>[study_stage] = '2' and [fluid_2(3)] = '1'		radio<br>1 Yes<br>2 Wish-list<br>3 No
	212	[og_bm_csf_9]<br><br>Show the field ONLY if:<br>[study_stage] = '2' and [fluid_2(3)] = '1'		radio<br>1 Yes<br>2 No

	213	[og_bm_csf_10]<br><br>Show the field ONLY if:<br>[study_stage] = '2' and [fluid_2(3)] = '1'		radio<br>1 Yes<br>2 No
	214	[og_bm_csf_o]<br><br>Show the field ONLY if:<br>[study_stage] = '2' and [fluid_2(3)] = '1' and [og_bm_csf_9] = '1'		text
	215	[og_bm_urine]<br><br>Show the field ONLY if:<br>[study_stage] = '2' and [fluid_2(4)] = '1'		radio<br>1 Yes<br>2 Wish-list<br>3 No
	216	[og_bm_urine_2]<br><br>Show the field ONLY if:<br>[study_stage] = '2' and [fluid_2(4)] = '1'		radio<br>1 Yes<br>2 Wish-list<br>3 No
	217	[og_bm_urine_3]<br><br>Show the field ONLY if:<br>[study_stage] = '2' and [fluid_2(4)] = '1'		radio<br>1 Yes<br>2 Wish-list<br>3 No
	218	[og_bm_urine_4]<br><br>Show the field ONLY if:<br>[study_stage] = '2' and [fluid_2(4)] = '1'		radio<br>1 Yes<br>2 Wish-list<br>3 No
	219	[og_bm_urine_5]<br><br>Show the field ONLY if:<br>[study_stage] = '2' and [fluid_2(4)] = '1'		radio<br>1 Yes<br>2 Wish-list<br>3 No
	220	[og_bm_urine_6]<br><br>Show the field ONLY if:<br>[study_stage] = '2' and [fluid_2(4)] = '1'		radio<br>1 Yes<br>2 Wish-list<br>3 No
	221	[og_bm_urine_7]<br><br>Show the field ONLY if:<br>[study_stage] = '2' and [fluid_2(4)] = '1'		radio<br>1 Yes<br>2 Wish-list<br>3 No
	222	[og_bm_urine_8]<br><br>Show the field ONLY if:<br>[study_stage] = '2' and [fluid_2(4)] = '1'		radio<br>1 Yes<br>2 Wish-list<br>3 No
	223	[og_bm_urine_9]<br><br>Show the field ONLY if:<br>[study_stage] = '2' and [fluid_2(4)] = '1'		radio<br>1 Yes<br>2 No
	224	[og_bm_urine_10]<br><br>Show the field ONLY if:<br>[study_stage] = '2' and [fluid_2(4)] = '1'		radio<br>1 Yes<br>2 No
	225	[og_bm_urine_o]<br><br>Show the field ONLY if:<br>[study_stage] = '2' and [fluid_2(4)] = '1' and [og_bm_urine_9] = '1'		text

	226	[og_bm_saliva]<br><br>Show the field ONLY if:<br>[study_stage] = '2' and [fluid_2(5)] = '1'		radio<br>1 Yes<br>2 Wish-list<br>3 No
	227	[og_bm_saliva_2]<br><br>Show the field ONLY if:<br>[study_stage] = '2' and [fluid_2(5)] = '1'		radio<br>1 Yes<br>2 Wish-list<br>3 No
	228	[og_bm_saliva_3]<br><br>Show the field ONLY if:<br>[study_stage] = '2' and [fluid_2(5)] = '1'		radio<br>1 Yes<br>2 Wish-list<br>3 No
	229	[og_bm_saliva_4]<br><br>Show the field ONLY if:<br>[study_stage] = '2' and [fluid_2(5)] = '1'		radio<br>1 Yes<br>2 Wish-list<br>3 No
	230	[og_bm_saliva_5]<br><br>Show the field ONLY if:<br>[study_stage] = '2' and [fluid_2(5)] = '1'		radio<br>1 Yes<br>2 Wish-list<br>3 No
	231	[og_bm_saliva_6]<br><br>Show the field ONLY if:<br>[study_stage] = '2' and [fluid_2(5)] = '1'		radio<br>1 Yes<br>2 Wish-list<br>3 No
	232	[og_bm_saliva_7]<br><br>Show the field ONLY if:<br>[study_stage] = '2' and [fluid_2(5)] = '1'		radio<br>1 Yes<br>2 Wish-list<br>3 No
	233	[og_bm_saliva_8]<br><br>Show the field ONLY if:<br>[study_stage] = '2' and [fluid_2(5)] = '1'		radio<br>1 Yes<br>2 Wish-list<br>3 No
	234	[og_bm_saliva_9]<br><br>Show the field ONLY if:<br>[study_stage] = '2' and [fluid_2(5)] = '1'		radio<br>1 Yes<br>2 No
	235	[og_bm_saliva_10]<br><br>Show the field ONLY if:<br>[study_stage] = '2' and [fluid_2(5)] = '1'		radio<br>1 Yes<br>2 No
	236	[og_bm_saliva_o]<br><br>Show the field ONLY if:<br>[study_stage] = '2' and [fluid_2(5)] = '1' and [og_bm_saliva_9] = '1'		text
	237	[ongoign_ab]	Abeta assay	notes
	238	[ongoign_ttau]	t-tau assay	notes
	239	[ongoign_ptau]	p-tau assay	notes
	240	[ongoign_gfap]	GFAP assay	notes
	241	[ongoign_nfl]	NfL assay	notes
	242	[ongoign_prot]	proteomics assay	notes
	243	[ongoign_metab]	metabolomics assay	notes
	244	[ongoign_lipi]	lipidomics assay	notes
	245	[ongoign_epi]	epigenomics assay	notes
	246	[ongoign_gwas]	GWAS assay	notes

	247	[ongoign_other]	other assay	notes
	248	[compl_biomarkers_in_fluids]<br><br>Show the field ONLY if:<br>[study_stage] = '3' and [collecting_3] = '1'	Please select the biomarkers measured in different biosamples in the completed project and the platform(s) you plan to use to analyze the different markers. Answer options: Yes (ongoing or planned), Wish-list (grant applications planned/pending), No β-Amyloid Total Tau Phosphorylated Tau Glial fibrillary acid protein GWAS Plasma {cm_bm_plasma} {cm_bm_plasma_2} {cm_bm_plasma_3} {cm_bm_plasma_4} Serum {cm_bm_serum} {cm_bm_serum_2} {cm_bm_serum_3} {cm_bm_serum_4} CSF {cm_bm_csf} {cm_bm_csf_2} {cm_bm_csf_3} {cm_bm_csf_4} Urine {cm_bm_urine} {cm_bm_urine_2} {cm_bm_urine_3} {cm_bm_urine_4} Saliva {cm_bm_saliva} {cm_bm_saliva_2} {cm_bm_saliva_3} {cm_bm_saliva_4} Whole blood {cm_bm_wb} Platform(s)* {compl_ab} {compl_ttau} {compl_ptau} {compl_gfap} {compl_gwas} *Please specify the sample type for each platform Neurofilament light Proteomics Metabolomics Epigenomics Lipidomics Other Plasma {cm_bm_plasma_10} {cm_bm_plasma_5} {cm_bm_plasma_6} {cm_bm_plasma_7} {cm_bm_plasma_8} {cm_bm_plasma_9} {cm_bm_plasma_o} Serum {cm_bm_serum_10} {cm_bm_serum_5} {cm_bm_serum_6} {cm_bm_serum_7} {cm_bm_serum_8} {cm_bm_serum_9} {cm_bm_serum_o} CSF {cm_bm_csf_10} {cm_bm_csf_5} {cm_bm_csf_6} {cm_bm_csf_7} {cm_bm_csf_8} {cm_bm_csf_9} {cm_bm_csf_o} Urine {cm_bm_urine_10} {cm_bm_urine_5} {cm_bm_urine_6} {cm_bm_urine_7} {cm_bm_urine_8} {cm_bm_urine_9} {cm_bm_urine_o} Saliva {cm_bm_saliva_10} {cm_bm_saliva_5} {cm_bm_saliva_6} {cm_bm_saliva_7} {cm_bm_saliva_8} {cm_bm_saliva_9} {cm_bm_saliva_o} Platform(s)* {compl_nfl} {compl_prot} {compl_lipi} {compl_metab} {compl_epi} {compl_other} *Please specify the sample type for each platform	descriptive
	249	[other_compl]<br><br>Show the field ONLY if:<br>[fluid(6)] = '1'	Please specify the biomarkers measured in other types of biosamples	text
	250	[cm_bm_wb]<br><br>Show the field ONLY if:<br>[study_stage] = '3' and [fluid(7)] = '1'		radio<br>1 Yes<br>2 Wish-list<br>3 No
	251	[cm_bm_plasma]<br><br>Show the field ONLY if:<br>[study_stage] = '3' and [fluid(1)] = '1'		radio<br>1 Yes<br>2 Wish-list<br>3 No
	252	[cm_bm_plasma_2]<br><br>Show the field ONLY if:<br>[study_stage] = '3' and [fluid(1)] = '1'		radio<br>1 Yes<br>2 Wish-list<br>3 No
	253	[cm_bm_plasma_3]<br><br>Show the field ONLY if:<br>[study_stage] = '3' and [fluid(1)] = '1'		radio<br>1 Yes<br>2 Wish-list<br>3 No
	254	[cm_bm_plasma_4]<br><br>Show the field ONLY if:<br>[study_stage] = '3' and [fluid(1)] = '1'		radio<br>1 Yes<br>2 Wish-list<br>3 No
	255	[cm_bm_plasma_5]<br><br>Show the field ONLY if:<br>[study_stage] = '3' and [fluid(1)] = '1'		radio<br>1 Yes<br>2 Wish-list<br>3 No

	256	[cm_bm_plasma_6]<br><br>Show the field ONLY if:<br>[study_stage] = '3' and [fluid(1)] = '1'		radio<br>1 Yes<br>2 Wish-list<br>3 No
	257	[cm_bm_plasma_7]<br><br>Show the field ONLY if:<br>[study_stage] = '3' and [fluid(1)] = '1'		radio<br>1 Yes<br>2 Wish-list<br>3 No
	258	[cm_bm_plasma_8]<br><br>Show the field ONLY if:<br>[study_stage] = '3' and [fluid(1)] = '1'		radio<br>1 Yes<br>2 Wish-list<br>3 No
	259	[cm_bm_plasma_9]<br><br>Show the field ONLY if:<br>[study_stage] = '3' and [fluid(1)] = '1'		radio<br>1 Yes<br>2 Wish-list<br>3 No
	260	[cm_bm_plasma_10]<br><br>Show the field ONLY if:<br>[study_stage] = '3' and [fluid(1)] = '1'		radio<br>1 Yes<br>2 Wish-list<br>3 No
	261	[cm_bm_plasma_o]<br><br>Show the field ONLY if:<br>[study_stage] = '3' and [fluid(1)] = '1' and [cm_bm_plasma_9] = '1'		text
	262	[cm_bm_serum]<br><br>Show the field ONLY if:<br>[study_stage] = '3' and [fluid(2)] = '1'		radio<br>1 Yes<br>2 Wish-list<br>3 No
	263	[cm_bm_serum_2]<br><br>Show the field ONLY if:<br>[study_stage] = '3' and [fluid(2)] = '1'		radio<br>1 Yes<br>2 Wish-list<br>3 No
	264	[cm_bm_serum_3]<br><br>Show the field ONLY if:<br>[study_stage] = '3' and [fluid(2)] = '1'		radio<br>1 Yes<br>2 Wish-list<br>3 No
	265	[cm_bm_serum_4]<br><br>Show the field ONLY if:<br>[study_stage] = '3' and [fluid(2)] = '1'		radio<br>1 Yes<br>2 Wish-list<br>3 No
	266	[cm_bm_serum_5]<br><br>Show the field ONLY if:<br>[study_stage] = '3' and [fluid(2)] = '1'		radio<br>1 Yes<br>2 Wish-list<br>3 No
	267	[cm_bm_serum_6]<br><br>Show the field ONLY if:<br>[study_stage] = '3' and [fluid(2)] = '1'		radio<br>1 Yes<br>2 Wish-list<br>3 No
	268	[cm_bm_serum_7]<br><br>Show the field ONLY if:<br>[study_stage] = '3' and [fluid(2)] = '1'		radio<br>1 Yes<br>2 Wish-list<br>3 No

	269	[cm_bm_serum_8]<br><br>Show the field ONLY if:<br>[study_stage] = '3' and [fluid(2)] = '1'		radio<br>1 Yes<br>2 Wish-list<br>3 No
	270	[cm_bm_serum_9]<br><br>Show the field ONLY if:<br>[study_stage] = '3' and [fluid(2)] = '1'		radio<br>1 Yes<br>2 Wish-list<br>3 No
	271	[cm_bm_serum_10]<br><br>Show the field ONLY if:<br>[study_stage] = '3' and [fluid(2)] = '1'		radio<br>1 Yes<br>2 Wish-list<br>3 No
	272	[cm_bm_serum_o]<br><br>Show the field ONLY if:<br>[study_stage] = '3' and [fluid(2)] = '1' and [cm_bm_serum_9] = '1'		text
	273	[cm_bm_csf]<br><br>Show the field ONLY if:<br>[study_stage] = '3' and [fluid(3)] = '1'		radio<br>1 Yes<br>2 Wish-list<br>3 No
	274	[cm_bm_csf_2]<br><br>Show the field ONLY if:<br>[study_stage] = '3' and [fluid(3)] = '1'		radio<br>1 Yes<br>2 Wish-list<br>3 No
	275	[cm_bm_csf_3]<br><br>Show the field ONLY if:<br>[study_stage] = '3' and [fluid(3)] = '1'		radio<br>1 Yes<br>2 Wish-list<br>3 No
	276	[cm_bm_csf_4]<br><br>Show the field ONLY if:<br>[study_stage] = '3' and [fluid(3)] = '1'		radio<br>1 Yes<br>2 Wish-list<br>3 No
	277	[cm_bm_csf_5]<br><br>Show the field ONLY if:<br>[study_stage] = '3' and [fluid(3)] = '1'		radio<br>1 Yes<br>2 Wish-list<br>3 No
	278	[cm_bm_csf_6]<br><br>Show the field ONLY if:<br>[study_stage] = '3' and [fluid(3)] = '1'		radio<br>1 Yes<br>2 Wish-list<br>3 No
	279	[cm_bm_csf_7]<br><br>Show the field ONLY if:<br>[study_stage] = '3' and [fluid(3)] = '1'		radio<br>1 Yes<br>2 Wish-list<br>3 No
	280	[cm_bm_csf_8]<br><br>Show the field ONLY if:<br>[study_stage] = '3' and [fluid(3)] = '1'		radio<br>1 Yes<br>2 Wish-list<br>3 No
	281	[cm_bm_csf_9]<br><br>Show the field ONLY if:<br>[study_stage] = '3' and [fluid(3)] = '1'		radio<br>1 Yes<br>2 Wish-list<br>3 No

	282	[cm_bm_csf_10]<br><br>Show the field ONLY if:<br>[study_stage] = '3' and [fluid (3)] = '1'		radio<br>1 Yes<br>2 Wish-list<br>3 No
	283	[cm_bm_csf_o]<br><br>Show the field ONLY if:<br>[study_stage] = '3' and [fluid (3)] = '1' and [cm_bm_csf_9] = '1'		text
	284	[cm_bm_urine]<br><br>Show the field ONLY if:<br>[study_stage] = '3' and [fluid (4)] = '1'		radio<br>1 Yes<br>2 Wish-list<br>3 No
	285	[cm_bm_urine_2]<br><br>Show the field ONLY if:<br>[study_stage] = '3' and [fluid (4)] = '1'		radio<br>1 Yes<br>2 Wish-list<br>3 No
	286	[cm_bm_urine_3]<br><br>Show the field ONLY if:<br>[study_stage] = '3' and [fluid (4)] = '1'		radio<br>1 Yes<br>2 Wish-list<br>3 No
	287	[cm_bm_urine_4]<br><br>Show the field ONLY if:<br>[study_stage] = '3' and [fluid (4)] = '1'		radio<br>1 Yes<br>2 Wish-list<br>3 No
	288	[cm_bm_urine_5]<br><br>Show the field ONLY if:<br>[study_stage] = '3' and [fluid (4)] = '1'		radio<br>1 Yes<br>2 Wish-list<br>3 No
	289	[cm_bm_urine_6]<br><br>Show the field ONLY if:<br>[study_stage] = '3' and [fluid (4)] = '1'		radio<br>1 Yes<br>2 Wish-list<br>3 No
	290	[cm_bm_urine_7]<br><br>Show the field ONLY if:<br>[study_stage] = '3' and [fluid (4)] = '1'		radio<br>1 Yes<br>2 Wish-list<br>3 No
	291	[cm_bm_urine_8]<br><br>Show the field ONLY if:<br>[study_stage] = '3' and [fluid (4)] = '1'		radio<br>1 Yes<br>2 Wish-list<br>3 No
	292	[cm_bm_urine_9]<br><br>Show the field ONLY if:<br>[study_stage] = '3' and [fluid (4)] = '1'		radio<br>1 Yes<br>2 Wish-list<br>3 No
	293	[cm_bm_urine_10]<br><br>Show the field ONLY if:<br>[study_stage] = '3' and [fluid (4)] = '1'		radio<br>1 Yes<br>2 Wish-list<br>3 No
	294	[cm_bm_urine_o]<br><br>Show the field ONLY if:<br>[study_stage] = '3' and [fluid (4)] = '1' and [cm_bm_urine_9] = '1'		text

	295	[cm_bm_saliva]<br><br>Show the field ONLY if:<br>[study_stage] = '3' and [fluid(5)] = '1'		radio<br>1 Yes<br>2 Wish-list<br>3 No
	296	[cm_bm_saliva_2]<br><br>Show the field ONLY if:<br>[study_stage] = '3' and [fluid(5)] = '1'		radio<br>1 Yes<br>2 Wish-list<br>3 No
	297	[cm_bm_saliva_3]<br><br>Show the field ONLY if:<br>[study_stage] = '3' and [fluid(5)] = '1'		radio<br>1 Yes<br>2 Wish-list<br>3 No
	298	[cm_bm_saliva_4]<br><br>Show the field ONLY if:<br>[study_stage] = '3' and [fluid(5)] = '1'		radio<br>1 Yes<br>2 Wish-list<br>3 No
	299	[cm_bm_saliva_5]<br><br>Show the field ONLY if:<br>[study_stage] = '3' and [fluid(5)] = '1'		radio<br>1 Yes<br>2 Wish-list<br>3 No
	300	[cm_bm_saliva_6]<br><br>Show the field ONLY if:<br>[study_stage] = '3' and [fluid(5)] = '1'		radio<br>1 Yes<br>2 Wish-list<br>3 No
	301	[cm_bm_saliva_7]<br><br>Show the field ONLY if:<br>[study_stage] = '3' and [fluid(5)] = '1'		radio<br>1 Yes<br>2 Wish-list<br>3 No
	302	[cm_bm_saliva_8]<br><br>Show the field ONLY if:<br>[study_stage] = '3' and [fluid(5)] = '1'		radio<br>1 Yes<br>2 Wish-list<br>3 No
	303	[cm_bm_saliva_9]<br><br>Show the field ONLY if:<br>[study_stage] = '3' and [fluid(5)] = '1'		radio<br>1 Yes<br>2 Wish-list<br>3 No
	304	[cm_bm_saliva_10]<br><br>Show the field ONLY if:<br>[study_stage] = '3' and [fluid(5)] = '1'		radio<br>1 Yes<br>2 Wish-list<br>3 No
	305	[cm_bm_saliva_o]<br><br>Show the field ONLY if:<br>[study_stage] = '3' and [fluid(5)] = '1' and [cm_bm_saliva_9] = '1'		text
	306	[compl_ab]	Abeta assay	notes
	307	[compl_ttau]	t-tau assay	notes
	308	[compl_ptau]	p-tau assay	notes
	309	[compl_gfap]	GFAP assay	notes
	310	[compl_nfl]	NfL assay	notes
	311	[compl_prot]	proteomics assay	notes
	312	[compl_metab]	metabolomics assay	notes
	313	[compl_lipi]	lipidomics assay	notes
	314	[compl_epi]	epigenomics assay	notes

	315	[compl_gwas]	GWAS assay	notes
	316	[compl_other]	other assay	notes
	317	[total_cholesterol]<br><br>Show the field ONLY if:<br>[fluid_2(7)] = '1' or [fluid_2(8)] = '1' or [fluid_2(9)] = '1' or [fluid_2(1)] = '1' or [fluid_2(2)] = '1' or [fluid_2(4)] = '1' or [fluid(7)] = '1' or [fluid(8)] = '1' or [fluid(9)] = '1' or [fluid(1)] = '1' or [fluid(2)] = '1' or [fluid(4)] = '1'	Section Header: *Which clinical laboratory markers are measured?*<br>Total cholesterol	checkbox<br>1 total_cholesterol___1 Blood<br>2 total_cholesterol___2 Urine
	318	[ldl_c]<br><br>Show the field ONLY if:<br>[fluid_2(7)] = '1' or [fluid_2(8)] = '1' or [fluid_2(9)] = '1' or [fluid_2(1)] = '1' or [fluid_2(2)] = '1' or [fluid_2(4)] = '1' or [fluid(7)] = '1' or [fluid(8)] = '1' or [fluid(9)] = '1' or [fluid(1)] = '1' or [fluid(2)] = '1' or [fluid(4)] = '1'	LDL-C	checkbox<br>1 ldl_c___1 Blood<br>2 ldl_c___2 Urine
	319	[hdl_c]<br><br>Show the field ONLY if:<br>[fluid_2(7)] = '1' or [fluid_2(8)] = '1' or [fluid_2(9)] = '1' or [fluid_2(1)] = '1' or [fluid_2(2)] = '1' or [fluid_2(4)] = '1' or [fluid(7)] = '1' or [fluid(8)] = '1' or [fluid(9)] = '1' or [fluid(1)] = '1' or [fluid(2)] = '1' or [fluid(4)] = '1'	HDL-C	checkbox<br>1 hdl_c___1 Blood<br>2 hdl_c___2 Urine
	320	[triglycerides]<br><br>Show the field ONLY if:<br>[fluid_2(7)] = '1' or [fluid_2(8)] = '1' or [fluid_2(9)] = '1' or [fluid_2(1)] = '1' or [fluid_2(2)] = '1' or [fluid_2(4)] = '1' or [fluid(7)] = '1' or [fluid(8)] = '1' or [fluid(9)] = '1' or [fluid(1)] = '1' or [fluid(2)] = '1' or [fluid(4)] = '1'	Triglycerides	checkbox<br>1 triglycerides___1 Blood<br>2 triglycerides___2 Urine
	321	[hba1c]<br><br>Show the field ONLY if:<br>[fluid_2(7)] = '1' or [fluid_2(8)] = '1' or [fluid_2(9)] = '1' or [fluid_2(1)] = '1' or [fluid_2(2)] = '1' or [fluid_2(4)] = '1' or [fluid(7)] = '1' or [fluid(8)] = '1' or [fluid(9)] = '1' or [fluid(1)] = '1' or [fluid(2)] = '1' or [fluid(4)] = '1'	HbA1c	checkbox<br>1 hba1c___1 Blood<br>2 hba1c___2 Urine
	322	[glucose]<br><br>Show the field ONLY if:<br>[fluid_2(7)] = '1' or [fluid_2(8)] = '1' or [fluid_2(9)] = '1' or [fluid_2(1)] = '1' or [fluid_2(2)] = '1' or [fluid_2(4)] = '1' or [fluid(7)] = '1' or [fluid(8)] = '1' or [fluid(9)] = '1' or [fluid(1)] = '1' or [fluid(2)] = '1' or [fluid(4)] = '1'	Glucose	checkbox<br>1 glucose___1 Blood<br>2 glucose___2 Urine
	323	[tsh]<br><br>Show the field ONLY if:<br>[fluid_2(7)] = '1' or [fluid_2(8)] = '1' or [fluid_2(9)] = '1' or [fluid_2(1)] = '1' or [fluid_2(2)] = '1' or [fluid_2(4)] = '1' or [fluid(7)] = '1' or [fluid(8)] = '1' or [fluid(9)] = '1' or [fluid(1)] = '1' or [fluid(2)] = '1' or [fluid(4)] = '1'	TSH	checkbox<br>1 tsh___1 Blood<br>2 tsh___2 Urine

	324	[t4]<br><br>Show the field ONLY if:<br>[fluid_2(7)] = '1' or [fluid_2(8)] = '1' or [fluid_2(9)] = '1' or [fluid_2(1)] = '1' or [fluid_2(2)] = '1' or [fluid_2(4)] = '1' or [fluid(7)] = '1' or [fluid(8)] = '1' or [fluid(9)] = '1' or [fluid(1)] = '1' or [fluid(2)] = '1' or [fluid(4)] = '1'	T4	checkbox<br>1 t4___1 Blood<br>2 t4___2 Urine
	325	[hemoglobin]<br><br>Show the field ONLY if:<br>[fluid_2(7)] = '1' or [fluid_2(8)] = '1' or [fluid_2(9)] = '1' or [fluid_2(1)] = '1' or [fluid_2(2)] = '1' or [fluid_2(4)] = '1' or [fluid(7)] = '1' or [fluid(8)] = '1' or [fluid(9)] = '1' or [fluid(1)] = '1' or [fluid(2)] = '1' or [fluid(4)] = '1'	Hemoglobin	checkbox<br>1 hemoglobin___1 Blood<br>2 hemoglobin___2 Urine
	326	[ferritin]<br><br>Show the field ONLY if:<br>[fluid_2(7)] = '1' or [fluid_2(8)] = '1' or [fluid_2(9)] = '1' or [fluid_2(1)] = '1' or [fluid_2(2)] = '1' or [fluid_2(4)] = '1' or [fluid(7)] = '1' or [fluid(8)] = '1' or [fluid(9)] = '1' or [fluid(1)] = '1' or [fluid(2)] = '1' or [fluid(4)] = '1'	Ferritin	checkbox<br>1 ferritin___1 Blood<br>2 ferritin___2 Urine
	327	[potassium]<br><br>Show the field ONLY if:<br>[fluid_2(7)] = '1' or [fluid_2(8)] = '1' or [fluid_2(9)] = '1' or [fluid_2(1)] = '1' or [fluid_2(2)] = '1' or [fluid_2(4)] = '1' or [fluid(7)] = '1' or [fluid(8)] = '1' or [fluid(9)] = '1' or [fluid(1)] = '1' or [fluid(2)] = '1' or [fluid(4)] = '1'	Potassium	checkbox<br>1 potassium___1 Blood<br>2 potassium___2 Urine
	328	[sodium]<br><br>Show the field ONLY if:<br>[fluid_2(7)] = '1' or [fluid_2(8)] = '1' or [fluid_2(9)] = '1' or [fluid_2(1)] = '1' or [fluid_2(2)] = '1' or [fluid_2(4)] = '1' or [fluid(7)] = '1' or [fluid(8)] = '1' or [fluid(9)] = '1' or [fluid(1)] = '1' or [fluid(2)] = '1' or [fluid(4)] = '1'	Sodium	checkbox<br>1 sodium___1 Blood<br>2 sodium___2 Urine
	329	[calcium]<br><br>Show the field ONLY if:<br>[fluid_2(7)] = '1' or [fluid_2(8)] = '1' or [fluid_2(9)] = '1' or [fluid_2(1)] = '1' or [fluid_2(2)] = '1' or [fluid_2(4)] = '1' or [fluid(7)] = '1' or [fluid(8)] = '1' or [fluid(9)] = '1' or [fluid(1)] = '1' or [fluid(2)] = '1' or [fluid(4)] = '1'	Calcium	checkbox<br>1 calcium___1 Blood<br>2 calcium___2 Urine
	330	[creatinine]<br><br>Show the field ONLY if:<br>[fluid_2(7)] = '1' or [fluid_2(8)] = '1' or [fluid_2(9)] = '1' or [fluid_2(1)] = '1' or [fluid_2(2)] = '1' or [fluid_2(4)] = '1' or [fluid(7)] = '1' or [fluid(8)] = '1' or [fluid(9)] = '1' or [fluid(1)] = '1' or [fluid(2)] = '1' or [fluid(4)] = '1'	Creatinine	checkbox<br>1 creatinine___1 Blood<br>2 creatinine___2 Urine

	331	[albumin]<br><br>Show the field ONLY if:<br>[fluid_2(7)] = '1' or [fluid_2(8)] = '1' or [fluid_2(9)] = '1' or [fluid_2(1)] = '1' or [fluid_2(2)] = '1' or [fluid_2(4)] = '1' or [fluid(7)] = '1' or [fluid(8)] = '1' or [fluid(9)] = '1' or [fluid(1)] = '1' or [fluid(2)] = '1' or [fluid(4)] = '1'	Albumin	checkbox<br>1 | albumin___1 | Blood<br>2 | albumin___2 | Urine
	332	[crp]<br><br>Show the field ONLY if:<br>[fluid_2(7)] = '1' or [fluid_2(8)] = '1' or [fluid_2(9)] = '1' or [fluid_2(1)] = '1' or [fluid_2(2)] = '1' or [fluid_2(4)] = '1' or [fluid(7)] = '1' or [fluid(8)] = '1' or [fluid(9)] = '1' or [fluid(1)] = '1' or [fluid(2)] = '1' or [fluid(4)] = '1'	CRP	checkbox<br>1 | crp___1 | Blood<br>2 | crp___2 | Urine
	333	[vitamin_d]<br><br>Show the field ONLY if:<br>[fluid_2(7)] = '1' or [fluid_2(8)] = '1' or [fluid_2(9)] = '1' or [fluid_2(1)] = '1' or [fluid_2(2)] = '1' or [fluid_2(4)] = '1' or [fluid(7)] = '1' or [fluid(8)] = '1' or [fluid(9)] = '1' or [fluid(1)] = '1' or [fluid(2)] = '1' or [fluid(4)] = '1'	Vitamin D	checkbox<br>1 | vitamin_d___1 | Blood<br>2 | vitamin_d___2 | Urine
	334	[vitamin_b12]<br><br>Show the field ONLY if:<br>[fluid_2(7)] = '1' or [fluid_2(8)] = '1' or [fluid_2(9)] = '1' or [fluid_2(1)] = '1' or [fluid_2(2)] = '1' or [fluid_2(4)] = '1' or [fluid(7)] = '1' or [fluid(8)] = '1' or [fluid(9)] = '1' or [fluid(1)] = '1' or [fluid(2)] = '1' or [fluid(4)] = '1'	Vitamin B12	checkbox<br>1 | vitamin_b12___1 | Blood<br>2 | vitamin_b12___2 | Urine
	335	[folic_acid]<br><br>Show the field ONLY if:<br>[fluid_2(7)] = '1' or [fluid_2(8)] = '1' or [fluid_2(9)] = '1' or [fluid_2(1)] = '1' or [fluid_2(2)] = '1' or [fluid_2(4)] = '1' or [fluid(7)] = '1' or [fluid(8)] = '1' or [fluid(9)] = '1' or [fluid(1)] = '1' or [fluid(2)] = '1' or [fluid(4)] = '1'	Folic Acid	checkbox<br>1 | folic_acid___1 | Blood<br>2 | folic_acid___2 | Urine
	336	[afos]<br><br>Show the field ONLY if:<br>[fluid_2(7)] = '1' or [fluid_2(8)] = '1' or [fluid_2(9)] = '1' or [fluid_2(1)] = '1' or [fluid_2(2)] = '1' or [fluid_2(4)] = '1' or [fluid(7)] = '1' or [fluid(8)] = '1' or [fluid(9)] = '1' or [fluid(1)] = '1' or [fluid(2)] = '1' or [fluid(4)] = '1'	AFOS	checkbox<br>1 | afos___1 | Blood<br>2 | afos___2 | Urine
	337	[alat]<br><br>Show the field ONLY if:<br>[fluid_2(7)] = '1' or [fluid_2(8)] = '1' or [fluid_2(9)] = '1' or [fluid_2(1)] = '1' or [fluid_2(2)] = '1' or [fluid_2(4)] = '1' or [fluid(7)] = '1' or [fluid(8)] = '1' or [fluid(9)] = '1' or [fluid(1)] = '1' or [fluid(2)] = '1' or [fluid(4)] = '1'	ALAT	checkbox<br>1 | alat___1 | Blood<br>2 | alat___2 | Urine

	338	[asat]<br>Show the field ONLY if:<br>[fluid_2(7)] = '1' or [fluid_2(8)] = '1' or [fluid_2(9)] = '1' or [fluid_2(1)] = '1' or [fluid_2(2)] = '1' or [fluid_2(4)] = '1' or [fluid(7)] = '1' or [fluid(8)] = '1' or [fluid(9)] = '1' or [fluid(1)] = '1' or [fluid(2)] = '1' or [fluid(4)] = '1'	ASAT	checkbox<br>1 asat___1 Blood<br>2 asat___2 Urine
	339	[leukocytes]<br>Show the field ONLY if:<br>[fluid_2(7)] = '1' or [fluid_2(8)] = '1' or [fluid_2(9)] = '1' or [fluid_2(1)] = '1' or [fluid_2(2)] = '1' or [fluid_2(4)] = '1' or [fluid(7)] = '1' or [fluid(8)] = '1' or [fluid(9)] = '1' or [fluid(1)] = '1' or [fluid(2)] = '1' or [fluid(4)] = '1'	Leukocytes	checkbox<br>1 leukocytes___1 Blood<br>2 leukocytes___2 Urine
	340	[erytrocytes]<br>Show the field ONLY if:<br>[fluid_2(7)] = '1' or [fluid_2(8)] = '1' or [fluid_2(9)] = '1' or [fluid_2(1)] = '1' or [fluid_2(2)] = '1' or [fluid_2(4)] = '1' or [fluid(7)] = '1' or [fluid(8)] = '1' or [fluid(9)] = '1' or [fluid(1)] = '1' or [fluid(2)] = '1' or [fluid(4)] = '1'	Erytrocytes	checkbox<br>1 erytrocytes___1 Blood<br>2 erytrocytes___2 Urine
	341	[trombocytes]<br>Show the field ONLY if:<br>[fluid_2(7)] = '1' or [fluid_2(8)] = '1' or [fluid_2(9)] = '1' or [fluid_2(1)] = '1' or [fluid_2(2)] = '1' or [fluid_2(4)] = '1' or [fluid(7)] = '1' or [fluid(8)] = '1' or [fluid(9)] = '1' or [fluid(1)] = '1' or [fluid(2)] = '1' or [fluid(4)] = '1'	Trombocytes	checkbox<br>1 trombocytes___1 Blood<br>2 trombocytes___2 Urine
	342	[hematocrit]<br>Show the field ONLY if:<br>[fluid_2(7)] = '1' or [fluid_2(8)] = '1' or [fluid_2(9)] = '1' or [fluid_2(1)] = '1' or [fluid_2(2)] = '1' or [fluid_2(4)] = '1' or [fluid(7)] = '1' or [fluid(8)] = '1' or [fluid(9)] = '1' or [fluid(1)] = '1' or [fluid(2)] = '1' or [fluid(4)] = '1'	Hematocrit	checkbox<br>1 hematocrit___1 Blood<br>2 hematocrit___2 Urine
	343	[other]<br>Show the field ONLY if:<br>[fluid_2(7)] = '1' or [fluid_2(8)] = '1' or [fluid_2(9)] = '1' or [fluid_2(1)] = '1' or [fluid_2(2)] = '1' or [fluid_2(4)] = '1' or [fluid(7)] = '1' or [fluid(8)] = '1' or [fluid(9)] = '1' or [fluid(1)] = '1' or [fluid(2)] = '1' or [fluid(4)] = '1'	Other	checkbox<br>1 other___1 Blood<br>2 other___2 Urine
	344	[other_clinlab]<br>Show the field ONLY if:<br>[other(1)] = '1' or [other(2)] = '1'	Please, specify other marker(s)	text
	345	[support]	Section Header: *Additional information*<br>Please provide any additional information you would like to add about your biorepository and/or biomarker analyses.	notes
	346	[biomarkers_and_biorepository_studies_complete]	Section Header: *Form Status*<br>Complete?	dropdown<br>0 Incomplete<br>1 Unverified<br>2 Complete
